# [SNG2], a prion form of Cut4/Apc1, confers non-Mendelian inheritance of heterochromatin silencing defect in fission yeast

**DOI:** 10.1093/nar/gkae1136

**Published:** 2024-11-20

**Authors:** Suman Sharma, Suchita Srivastava, Rudra Narayan Dubey, Poonam Mishra, Jagmohan Singh

**Affiliations:** Institute of Microbial Technology, Sector 39A, Chandigarh 160036, India; Institute of Microbial Technology, Sector 39A, Chandigarh 160036, India; Institute of Microbial Technology, Sector 39A, Chandigarh 160036, India; Institute of Microbial Technology, Sector 39A, Chandigarh 160036, India; Institute of Microbial Technology, Sector 39A, Chandigarh 160036, India

## Abstract

Prions represent epigenetic regulator proteins that can self-propagate their structure and confer their misfolded structure and function on normally folded proteins. Like the mammalian prion PrP^Sc^, prions also occur in fungi. While a few prions, like Swi1, affect gene expression, none are shown to affect heterochromatin structure and function. In fission yeast and metazoans, histone methyltransferase Clr4/Suv39 causes H3-Lys9 methylation, which is bound by the chromodomain protein Swi6/HP1 to assemble heterochromatin. Earlier, we showed that *sng2-1* mutation in the Cut4 subunit of anaphase-promoting complex abrogates heterochromatin structure due to defective binding and recruitment of Swi6. Here, we demonstrate that the Cut4p forms a non-canonical prion form, designated as [SNG2], which abrogates heterochromatin silencing. [SNG2] exhibits various prion-like properties, e.g. non-Mendelian inheritance, requirement of Hsp proteins for its propagation, *de novo* generation upon *cut4* overexpression, reversible curing by guanidine, cytoplasmic inheritance and formation of infectious protein aggregates, which are dissolved upon overexpression of *hsp* genes. Interestingly, [SNG2] prion imparts an enhanced tolerance to stress conditions, supporting its role in promoting cell survival under environmental stress during evolution.

## Graphical Abstract

**Graphical Abstract:**
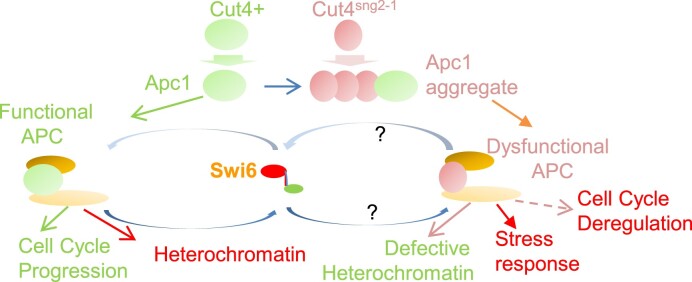

## Introduction

Following the discovery of prions by Prusiner (1) and their role in transmission of disease traits in humans and cows (2–5), work in the budding yeast *Saccharomyces cerevisiae* has validated the protein-based mechanism of inheritance. It has also provided a genetic system for deciphering the mechanistic details and ushered the possibility of developing screens for prion treatment. In *S. cerevisiae*, reinvestigation of the phenomena of non-Mendelian inheritance of some mutations reported earlier (6–10), led Wickner to provide evidence in support of protein-based inheritance of [URE3], hence named ‘prion’ (11). Wickner and others further showed that the [URE3] prion could be generated *de novo* upon overexpression of *URE2* gene in wild type (WT) but not in *ure2Δ* mutants and was cured by growth in presence of guanidine hydrochrloride (11–13). Subsequently, several new prions were discovered, such as, Sup35 with the prion form [PSI^−^] (14); [PIN], the prion form of Rnq1, which is required for *de novo* appearance of [PSI^+^] (15,16) and contains a Q/N-rich domain, that can confer prion function to Sup35 (17); Cyc8, with a Lac^+^ form [OCT^+^] (18) and Swi1 [*SWI^+^*] (19) in *S. cerevisiae* and [Het-s] in Podospora (20). In general, the prion form exhibited loss of function and displayed protein-based inheritance by virtue of its property to convert the normal protein structure into the prion form. As a result, the prion form shows dominance and cytoplasmic inheritance. Because of the dominant nature, the loss of function of the prion form generally leads to dominant negative effect with respect to the WT protein function (21). However, in a few cases the prion form of the protein is mooted to perform normal physiological function or shows gain of function, such as, Het-s in *Podospora* (20).

So far, only a few cases of prion proteins regulating gene expression have been reported. Among them Swi1, a component of the SWI-SNF yeast chromatin remodeling complex, regulates HO gene expression, Ty insertions etc. (19). Similarly, [OCT^+^], the prion form of transcriptional co-repressor Cyc8, causes functional inactivation of the Cyc-Tup1 complex, profoundly affecting global gene expression and cellular phenotypes, including defects in mating, sporulation etc. (18). However, there is no report showing the role of prion in regulation of heterochromatin structure and gene silencing. Rather, chromatids assembled into heterochromatin structure has been shown to segregate as Mendelian alleles (22).

Heterochromatin assembly in fission yeast occurs through an evolutionarily conserved pathway (23). According to one model, freshly assembled hyperacetylated histones are first deacetylated at H3-Lys9 position, followed by methylation by the H3-K9-methyltransferase Clr4/Suv39. H3-K9-Me2/Me3 serves as a binding site for the conserved chromodomain protein Swi6/HP1 (24). Further multimerization of Swi6/HP1, together with the cooperative interaction with Clr4, helps to extend the region occupied by the heterochromatin mark (H3-K9-Me2/Me3) and Swi6/HP1 in the flanking regions (25–27).

RNAi machinery also plays a role in silencing, particularly at the stage of establishment of the heterochromatin mark, H3K9-Me, but not in propagation in fission yeast (28). The hallmark of both the heterochromatin and RNAi pathways is their action at the chromatin level: the chromatin structure with its memory acts in a manner similar to a Mendelian allele (22) and does not display cytoplasmic regulation.

Earlier, we had reported the role of Cut4, the largest subunit of the anaphase-promoting complex (APC; 29), in heterochromatin silencing (30). The mutant allele *sng2-1*/*cut4* shows silencing defect, which exhibited semi-dominance (30), unlike the earlier reported recessive mutants, like *clr1-4*, *swi6*, etc. (31–33). Surprisingly, the silencing defect persisted even after the *sng2-1* mutation was outcrossed. Further analysis revealed another property of the encoded protein Cut4: it acts as a prion, which causes loss of silencing at the heterochromatin regions – mating type and centromere. The trait of silencing defect is inherited stably during multiple mitotic divisions and in a non-Mendelian manner during meiosis in a genetic cross with a WT strain. Accordingly, we tested whether this phenotype of silencing defect shows the known characteristic of prions and show that the silencing defect caused by the putative prion form of Cut4 is inherited in a non-Mendelian fashion, is cured by guanidine and overexpression of *hsp104* and *hsp70*, is dependent on *hsp104* for its generation and exhibits dominance and cytoplasmic inheritance along with protein aggregation. Importantly, the prion form of Cut4 also shows protein infectivity. Interestingly, the prion derivatives phenocopy the *sng2-1* and *cut4-533* mutants and exhibit enhanced tolerance to various stresses.

## Materials and methods

### Strains and plasmids

The parental strains used in most of the studies include SPA236 (genotype I: *mat1Msmto REII*Δ*mat2P*::*ura4 ura4D18 leu1-32 ade6-216*) and SPA302 (genotype II: *mat1PΔ17::leu2 REIIΔmat2P::ura4 leu1-32 ura4D18 his2^−^ ade6-210)*. The *sng2-1* mutation was transferred to parental strain SPA236 by crossing and checked for silencing effects. Mutations were transferred into the reporter strains by standard genetic crosses. The strains and plasmids used in this study are listed in Supplementary Tables S1 and S2 (Supplementary Data). Media, growth conditions and transformations were as described (34).

### Silencing phenotypes

Normal homothallic strains, denoted as *h^90^* switch at 90% efficiency between Plus and Minus mating types. These cells mate with each other to form zygotes, which undergo sporulation under starvation conditions. The spores have a starchy cell wall which gives purple staining with iodine. Thus, cells of efficiently switching *h^90^* strains form colonies that give dark staining with iodine and the phenotype is referred to as spo^+^. In contrast poorly switching mutants, like *swi6^−^*, form colonies that give poor staining with iodine, referred to as spo^−^ phenotype (31,34).

In contrast, non-switching WT strains with the genotype *mat1Msmto REIIΔ mat2P::ura4*, having a *mat2*-linked *ura4* reporter, have a silent *mat2* locus and give a spo^−^-ura^−^ phenotype. However, in the *swi6^−^* mutant background, they show a spo^+^-ura^+^ phenotype, with colonies giving dark staining with iodine, because the derepression of the silent *mat2Pc* locus, together with Minus information from *mat1M* locus, triggers meiosis, albeit in haploid cells, producing spores with starchy cell wall, which gives dark staining with iodine.

The cross to follow the nature of inheritance of the silencing defect requires the use of cells of opposite mating type. Since the *sng2-1* mutation elicits the spo^+^, ura^+^ phenotype in the strain with genotype I having Minus mating type (*mat1Msmto*, with linked *mat2* locus with Plus mating type and the *ura4* reporter), it was crossed with a strain with opposite mating type (*mat1PΔ17::LEU2*) and similar linked markers, denoted as genotype II.

It is well established that simultaneous expression of transcripts of Minus and Plus alleles triggers meiosis and sporulation in *Schizosaccharomyces pombe*. In wild-type heterothallic strain having *mat1* Minus locus, the donor loci *mat2P* (having Plus alleles) and *mat3* (having Minus alleles) are silent and the *mat2*-linked *ura4* reporter is silent. However, silencing defect of *mat2* locus, having a deletion of silencer (*REIIΔ*) caused by mutations like *swi6*, *clr1-clr4*, etc., leads to loss of silencing of the *mat2* locus. This triggers simultaneous expression of Minus alleles (from *mat1M* locus) and Plus alleles from *mat2P* locus and the *mat2P*-linked *ura4* reporter, leading to sporulation and the phenotypes: spo^+^, ura^+^. Similarly, the strain having a stable Plus allele at the *mat1* locus, designated *mat1PΔ17::LEU2* along with *mat2P::ura4* and *REIIΔ*, as above but an intact *mat3* locus, only expressed Plus allele at *mat1* locus, while Plus alleles at *mat2* and Minus alleles at *mat3* locus are silent. Thus, the Minus strain *mat1Msmto* above, and the strain with genotype *mat1PΔ17::LEU2* also show normal silencing of *mat2P* or *mat2P*-linked *ura4* locus. However, in the background of silencing mutation, the *mat2P* locus with *REIIΔ* and linked *ura4* reporter gets derepressed and leads to elevated *ura4* expression. Importantly, no spo^+^ phenotype is observed, indicating that only Plus alleles (from *mat1* and *mat2* loci) are expressed, while Minus allele from *mat3M* locus is not.

The loss of silencing can also happen in a diploid state, like in M/M diploid, with genotype: *mat1Msmto REIIΔ mat2P::ura4*/*mat1Msmto REIIΔ mat2P::ura4;* in combination with the homozygous mutation *swi6^−^/swi6^−^*, leading to formation of azygotic asci. This property was exploited in the cytoduction experiment (see the ‘Results’ section).

### Mapping of *sng2-1* mutation to *cut4* gene

The *sng2-1* mutation was mapped to *cut4* gene on basis of the following criteria:

- Complementation of the ts^−^ phenotype of *sng2-1* mutation by *cut4* gene.
- We crossed *sng2-1* and *cut4-533* mutants and dissected 35 tetrads. No ts^+^ segregants were obtained, confirming that *sng2-1* and *cut4-533* mutants were tightly linked.
- We crossed the *sng2-1* mutant with a WT strain and isolated DNA from five independent ts^−^ segregants. Sequencing showed that all five segregants contained the mutation at 984th position converting codon GTT (encoding Valine) to GCT (encoding Alanine).

### Plasmid construction

The genes in *S. pombe* were chromosomally deleted and tagged using different heterologous modules. Strains carrying deletion of *swi6*, *clr3*, *hsp104* and *tht1* were constructed using pFA6a-KanMX6 plasmids containing *kan^r^* module. The green fluorescent protein (GFP)-tagged *cut4* cassette was generated using the tagging vector pFA6a-GFP-KanMX6 (35). The *YFP-FLAG-(His)_6_* tagged *cut4* (YFH-*cut4*) strain was generated by integrating the YFH- *cut4* at *leu1* site by digesting the plasmid pYFH-*Cut4* with ApaI (procured from Riken DNA Bioresource Center (36).

The *tht1Δ* homozygous diploid was generated by protoplast fusion using inter-allelic complementation between *ade6-216* and *ade6-210* alleles (34,37).

### Determination of the rate of switching of the epigenetic states

We followed the method of Kipling *et al*. (38) to determine the rate of switching.

Cells of colonies exhibiting a particular epigenetic state (e.g. dark or spo^+^, light or spo^−^, ura^+^, ura^w^ or ura^−^) were grown in culture medium and after overnight growth, a known dilution was spread on suitable plate to score the phenotype. Remaining cells were inoculated into fresh medium at cell density of 10^5^ cells/ml and allowed to grow at 30°C for 15–20 generations, after which the same number of cells were spread on the same specific plate as before. After 3–4 days’ growth, number of colonies showing ura^+^ or spo^+^ phenotype were counted. The rate of switching was determined using the formula:


\begin{equation*}{\mathrm{Rate}} = 1 - {\left( {{\mathrm{F}}/{\mathrm{I}}} \right)^{1/{\mathrm{N}}}},\end{equation*}

where F is the final percentage of cells having spo^+^ or spo^−^, or ura^+^ or ura^−^ phenotype, I is the initial percentage of cells having spo^+^ or spo^−^, or ura^+^ or ura^−^ phenotype, and N is the number of generations.

### Visualization of analysis of prion aggregates and ‘cut’ phenotype

Cells expressing YFP and GFP-tagged *Cut4*p were visualized by confocal microscopy (Leica SP8 Confocal microscope). For visualizing ‘cut’ phenotype, cells were fixed with 70% ethanol, dehydrated, stained with 4'6-diamidino-2-phenylindole/ propitium iodide (DAPI/PI) and visualized under fluorescence microscope. For septum visualization calcofluor staining of the cells was followed by visualization under fluorescence microscope at 433 nm (34). For quantitation of cells containing YFP or GFP-Cut4 aggregates, number of cells containing fluorescent foci out of a total of 200 cells were counted.

### Curing by guanidine hydrochloride

Cells were grown in yeast extract with adenine (YEA) or selective medium with or without 4 mM GuHCl for 5–10 generations. An aliquot of GuHCl-treated cells was washed twice with sterile water and then grown in absence of GuHCl medium for five generations. Equal number of cells were taken from each sample, 10-fold serial dilutions were prepared and 2 μl of each dilution was spotted on complete, ura^−^ and 5-fluoroorotic acid (5-FOA) plates. For quantitative analysis, 50 μl of these cells at OD_600_ of 0.05 were plated on complete, ura^−^ and FOA plates.

### Curing of prion forms by overexpression of *hsp104* and *hsp70*

*hsp104* gene of *S. pombe* and *hsp70* gene of *S. cerevisae* were cloned in REP3X vector and transformed into ts^+^-ura^+^ segregants. Equal number of these transformants was plated on leu^−^ ura^−^ plates and ura^+^ cells were counted.

### Thermotolerance assay

Thermotolerance assay was carried out according to Eaglestone *et al.* (39) with minor changes. Two ura^+^-ts^+^ and two ura^−^-ts^+^ segregants were grown at 30°C to mid log phase in rich medium (OD_600_ = 0.4) and then transferred to 37°C for 1 h prior to heat treatment. Cells were diluted ∼3.5 × 10^3^-fold and transferred to a 52°C shaking water bath. Aliquots were removed at 5 min intervals and stored on ice. From these aliquots 150 μl (∼500 cells) were spread in triplicate onto YEA plate and colonies were counted after 4–5 days of growth at 30°C.

### Ethanol tolerance assay

Ethanol tolerance assay was performed according to Eaglestone *et al.* (39). Two ts^+^-ura^+^and two ts^+^-ura^−^ segregants were taken from freshly grown plate, resuspended into 500 μl of sterile water and diluted to 1 × 10^6^ cells/ml. Stress agar plate was prepared by adding 10% ethanol to YEA medium 24h prior to use. The stress agar plate was allowed to set with the plate tilted, so that there was no stress agar at one end of the plate. Just before use the same volume of YEA devoid of EtOH was poured on to stress agar and allowed to set. Then, 2 μl of cell suspension was spotted on to the stress agar plate along the gradient and sealed with parafilm to prevent evaporation of ethanol and then incubated at 30°C.

### Assay for heavy metal tolerance

Heavy metal tolerance assay was performed according to Yamashita *et al.* (29). Cells of prion and non-prion derivatives were taken from freshly grown plate, resuspended into 500 μl of sterile water and diluted to 1 × 10^6^ cells/ml. Chemostress agar plates containing concentrations of CdCl_2_, ranging from 1 to 20 μM, were prepared. A total of 2 μl of cell suspension was spotted on to the stress agar plate and then incubated at 30°C.

### Oxidative stress tolerance

Cells grown to the mid-log phase were subjected to H_2_O_2_ treatment. An overnight culture in 10 ml YEA medium was grown up to cell density of *∼*2 *×*10^7^ cells/ml (approximately 12th hr of culture growth). Cells were harvested. Test groups containing 0.2mM, 1.0 mM or 2.0 mM H_2_O_2_ and the control group without H_2_O_2_ were resuspended in 10 ml of YEA medium at the cell density of ∼1 × 10^7^ cells/ml and incubated at 30°C in a rotary shaker at 180 rpm for 30 min. To calculate cell viability, appropriate dilutions of the cultures were spread on YEA plates. These were incubated at 30°C and number of colonies were counted (40).

### Semi-denaturing detergent agarose gel electrophoresis

Overnight grown 25ml cultures were harvested, washed with distilled water and centrifuged at 2000 rcf (relative centrifugal field) for 5 min at room temperature. The cells were resuspended in 10 ml of spheroplasting solution having 1.2 M D-Sorbitol, 0.5 mM MgCl_2_, 20 mM Tris.HCl (pH 7.5), 50 mM β-mercaptoethanol and 0.5 mg/ml Zymolyase and incubated for 30 min at 30°C. The cells were checked for spheroplasting under microscope with 1% sodium dodecyl sulfate (SDS). The pellet was resuspended in lysis solution consisting of 25 mM Tris-HCl (pH 7.5), 15 mM MgCl_2_, 15 mM ethylene glycol-bis (β-aminoethyl ether)-N,N,N′,N′-tetraacetic acid (EGTA), 1 mM dithiothreitol (DTT), 60 mM β-glycerophosphate, 15 mM p-NPP, 0.5 mM sodium vanadate, 0.1 mM sodium fluoride and 1 mM phenylmethylsulfonyl fluoride (PMSF). Samples were lysed by vortexing at high speed for 5 min. The cellular debris was pelleted at 4000 rcf for 5 min. The supernatant was removed to a fresh tube. Samples were treated at room temperature for 7 min in a 4× sample buffer to give a final concentration of 2% sarkosyl, 5% glycerol, 0.5% TAE (Tris-Acetate-EDTA), 0.1 mg/ml bromophenol blue plus protease inhibitor cocktail (41). Electrophoresis of the samples was performed in 1.5% agarose gel containing 0.1% SDS (42). After electrophoresis, gels were subjected to western blotting with anti-GFP antibody.

### Protein infection

For the protein infection study, a standard protocol was used (43) with some modifications. We prepared the extracts from the prionogenic strains by the spheroplasting method, as reported by Tanaka *et al.* (44) and King and Diaz-Avalos (45). Cell pellet was resuspended in 400 μl of lysis buffer, as mentioned above. A total of 50 μl glass beads (0.5 mm, Sigma) were added to break cells in a bead beater for five 40 s cycles with samples being left on ice for 2 min between cycles. Cell debris was removed by centrifugation at 5000 rpm for 5 min at 4°C and the supernatant (‘total protein extract’) was centrifuged at 20 000 × *g* for 45 min to separate soluble from insoluble fractions. The pellet (insoluble fraction) was then re-suspended in 60 μl of lysis buffer. A total of 20 μl of insoluble fraction was treated with 0.025 units/μl benzonase to digest any nucleic acids present in the sample. Then, 20 ml of exponential phase cell culture of the recipient cells was processed for protein transformation according to Tanaka *et al.* (44) and the transformants were selected on pombe minimal adenine lacking leucine (PMA-leu) plates. Cell fractionation of normal and derivatives into soluble and pellet fractions was performed according Dulle *et al.* (46).

### Prion domain prediction software

We used the PSI-PRED and Disopred 3 softwares on the Protein Structure Prediction Server PSIPRED of the UCL Department of Computer Science: Bioinformatics Group, we predicted the features of secondary structure and potential to form Intrinsically disordered domains, which are generally correlated with the potential to form amyloid structure (47).

## Results

### *sng2-1*, a *cut4* allele with dominant negative effect on silencing, exhibits two epigenetic states

**Figure 1.**
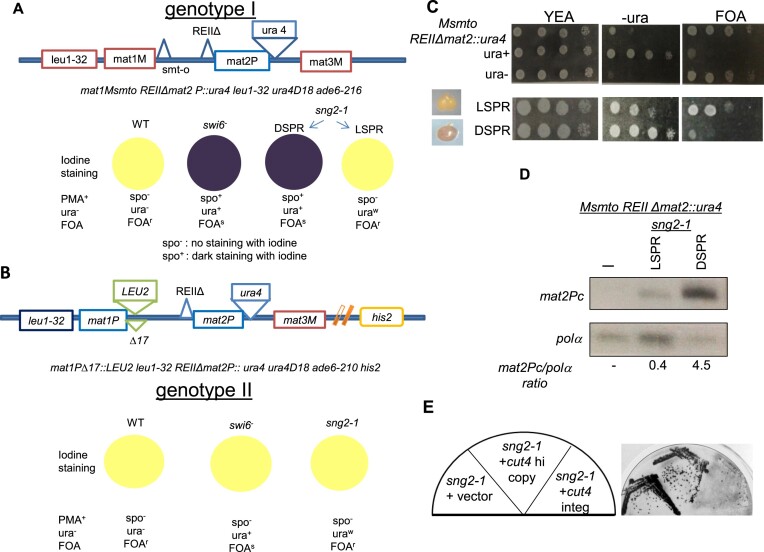
Two alternative silencing defective states displayed by the *sng2-1*/*cut4* mutant. (**A**) shows the genotype I, denoted as *leu1^−^mat1Msmto REIIΔmat2P::ura4* along with the associated phenotypes in the wt, *swi6^−^* and *sng2-1* background, giving the respective iodine staining phenotypes of the colonies on minimal media (PMA^+^): spo^−^, as light staining and spo^+^ representing dark staining. Also shown are the respective phenotypes of growth on media lacking uracil as ura^−^ or ura^+^, FOA^r^ or FOA^s^. (**B**) Genotype II, denoted as *mat1PΔ17::LEU2, REIIΔmat2P::ura4*, with a linked *his2^−^* marker. Also shown are the phenotypes associated with WT, *swi6^−^* and *sng2-1* backgrounds. (**C**) Spotting assay to assess the growth pattern of the control strain with genotype I along with control ura^+^ and ura^−^ strains on indicated plates. Also shown are the spo^−^ (LSPR) and spo^+^ (DSPR) derivatives of the *sng2-1* mutant with genotype I. (**D**) RT PCR analysis to monitor the level of the *polα* and *mat2Pc* transcripts in WT (-) and LSPR and DSPR derivatives of the *sng2-1* mutant. (**E**) Iodine phenotypes of the *sng2-1* mutant in background of genotype I transformed with vector, *cut4* gene in high copy vector or an integrating vector.

Earlier, we reported that a ts mutant, *sng2-1*, that grew well at 25°C and 30°C but failed to grow at 36°C, exhibited silencing defect at the mating type and centromere loci in fission yeast (30). The mutation was mapped to *cut4* gene (Materials and Methods) In the strain background *leu1^−^Msmto REIIΔmat2::ura4 ura4D18* (strain name SPA236; genotype I) with *Msmto* representing the unswitchable Minus allele at the *mat1* locus, *REIIΔ* representing the deletion of the repression element *REII* linked to the *mat2P* locus and *ura4* representing the *ura4* reporter gene inserted centromere distally to the *mat2P* locus) (32,33) (Figure 1A), the *sng2-1* mutant exhibited two metastable states with distinct phenotypes: (i) D (Dark or spo^+^, staining dark purple with iodine, termed as DSPR, due to loss of silencing) and ura^+^ (representing strong growth on plates lacking uracil) due to derepression of *ura4* reporter and (ii) L (light, spo^−^ giving no staining with iodine, termed as LSPR, like the parent strainSPA236) and ura^w^ (representing weaker growth on uracil lacking plates). In the strain background with the genotype: leu1−mat1PΔ17::LEU2 leu1-32 REIIΔ mat2::ura4, his2−, denoted as genotype II, which is similar to the genotype I except the mat1 locus harbors the Plus allele along with a linked deletion called Δ17 as well as insertion of the LEU2 reporter located centromere distally and linked to his2− marker (48), the sng2-1 mutant exhibits spo−-ura+ phenotype (Figure 1B). The differential growth on uracil lacking media in LSPR and DSPR is due to partially derepressed *ura4* reporter (34) (Figure 1C). A reciprocal growth level was also observed on FOA plates: ura^+^ cells grow poorly on FOA plates (FOA^s^), while ura^−^ cells grow well on FOA plates (FOA^r^) (Figure 1C). This was accompanied in the strain with the genotype I by strong expression of the silent copy transcript *mat2Pc* in the DSPR and weaker expression in the LSPR, but no expression in the WT parent (Figure 1D). The two states were metastable, switching to the opposite state at the rate of ∼10^−4^/generation during mitosis (Supplementary Figure S1B). Interestingly, the spo^+^ phenotype of the *sng2-1* mutant in the genotype I could not be complemented by the *cut4* gene on high copy vector and only partially by *cut4* gene on integrating vector, as indicated by iodine staining of the transformant colonies (Figure 1E). We think that the reason is as follows the single copy plasmid has the *cut4* gene cloned with its own promoter, while that of high copy plasmid has the *cut4* gene is expressed under the control of the thiamine-repressible promoter *nmt41*. Under this scenario, the *cut4* gene expressed under its own promoter is expressed under the normal physiological conditions and can complement the *sng2-1* mutation while that under the *nmt41* promoter is independent of normal physiological constraints and cannot overcome the dominant nature of the mutation. In contrast, the parent WT strain with genotype I does not give any iodine staining and thus shows spo^−^ phenotype (not shown). This indicates that the silencing defect of the *sng2-1* mutant was dominant or semi-dominant, unlike the recessive defect in the canonical silencing mutants *swi6*, *clr1-4*, etc. (31–33).

### The spo^+^- ura^+^ phenotype persists after outcrossing the *sng2-1* mutation

**Figure 2.**
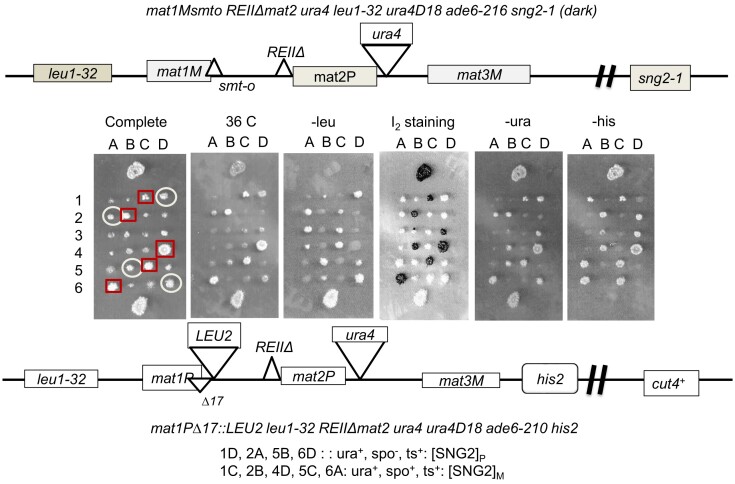
Persistence of the derepressed phenotype caused by the *sng2-1* mutation after outcrossing. Tetrad analysis shows the cross between the DSPR strain with genotype I (*leu1^−^ mat1Msmto REIIΔmat2P::ura4, ura4D18, sng2-1*) with a WT strain having genotype II (*leu1^−^ mat1PΔ17::LEU2, leu1-32, REIIΔmat2P::ura4, ura4D18, his2^−^*). Segregants listed as putative ‘[SNG2]_M_’ prion form are shown in square boxes and those as putative prion form ‘[SNG2]_P_^’^ are circled.

To further understand the dominant nature of the silencing defect, the *sng2-1* mutant in the genotype I with spo^+^ and ura^+^ phenotype (DSPR) was crossed with a WT strain having genotype II (Figure 2). WT strains in both genotypes I and II give spo^−^-ura^−^ phenotype, while the *sng2-1* mutant gives two inter-switching phenotypes of spo^+^-ura^+^ (DSPR) and spo^−^-ura^w^ (LSPR) in genotype I, as mentioned above, and spo^−^-ura^+^ phenotype in genotype II background (Figure 1A and B) (+ and – represented the strong and negative phenotypes, respectively). Interestingly, confocal microscopy showed that cells overexpressing GFP-tagged *cut4* gene on high copy vector showed punctate pattern, while cells harboring empty vector or those expressing truncated *cut4* gene (*cut4DB/B*) showed only a faint fluorescence (Figure 4F). Surprisingly, tetrad analysis of the cross between the strain DSPR and WT strain having genotype II showed that several ts^+^ segregants with genotype I (segregants 1C, 2B, 4D, 5C and 6A, in which the *sng2-1* mutation was outcrossed) showed spo^+^-ura^+^ phenotype (Figure 2). Likewise, several ts^+^ segregants (1D, 2A, 5B, 6D) with genotype II also showed ura^+^ phenotype (Figure 2). These results indicate that the phenotype engendered by the *sng2-1* mutation persisted even after it was outcrossed.

**Figure 3.**
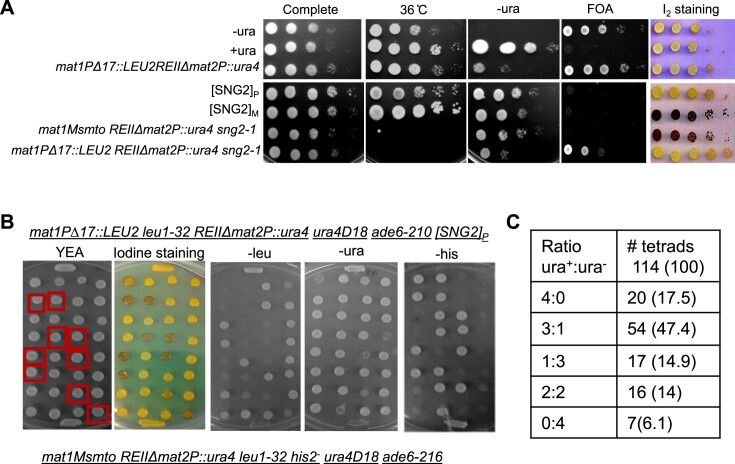
Non-Mendelian segregation of the putative [SNG2] prion form (**A**) Phenotypes of the wt, *sng2-1* mutant and the putative prion derivatives, [SNG2]_M_ and [SNG2]_P_, in the genetic backgrounds I and II, respectively, was monitored by spotting assay on indicated plates. (**B**) Tetrads derived from a cross of [SNG]_P_ strain having spo^−^-ura^+^ phenotype (top) and a WT strain (genotype I) having spo^−^-ura^−^ phenotype (bottom), were spotted on YEA and then replica plated to different plates, as indicated. Segregants representing the putative ‘[SNG2]_M_’ prion form are shown in square boxes. (**C**) Segregation pattern of ura^+^ phenotypes of the *mat2P::ura4* locus in tetrad analysis as shown in (**B**).

We denoted the leu^−^-ts^+^-spo^+^-ura^+^ segregants with genotype I as putative ‘[SNG2]_M_’ and those with genotype II with phenotype of leu^+^-spo^−^-ura^+^-his^−^ as putative ‘[SNG2]_P_^’^ prion forms, where M and P represent the mating types *mat1M* and *mat1P*, respectively, in which the latter is linked with *LEU2* and *his2*^−^ markers (Figures 2 and 3A).

We further tested whether the states ‘[SNG2]_P_’ and ‘[SNG2]_M_’ exhibit the known characteristics of prions.

### Prion form ‘[SNG2]_P_^’^ propagates in a non-Mendelian manner

To further investigate the possibility that the ura^+^ segregants display the properties of prions, we tested the pattern of inheritance of the ura^+^ phenotype by back-crossing cells of the putative ‘[SNG2]_P_’ prion form (from Figure 2) with WT cells of genotype I (strain SPA236). Interestingly, while both the parent strains show spo^−^ phenotype, we observed several spo^+^ segregants (indicated by boxes, Figure 3B). In addition, we observed the ura^+^: ura^−^ segregation pattern deviating from the Mendelian ratio of 2:2, with a majority of tetrads (∼86%) showing 4:0, 3:1, 1:3 and 0:4 segregation pattern and only 14% tetrads showing the 2:2 segregation pattern (see -ura panel. Figure 3B and C). This pattern of inheritance is similar to that displayed by the [URE3] prion (11). These results confirm the non-Mendelian pattern of segregation of the derepressed state, which was engendered by the *sng2-1*/*cut4^−^* mutation and persisted even after the mutation was outcrossed.

More tellingly, we found that among the 104 ts^+^ segregants of a back cross of *sng2-1* mutant with a WT strain, 64 (61.5%) were ura^+^ (representing the derepressed *mat2P* and the linked *ura4* locus), as against 0% expected by Mendelian segregation (Supplementary Figure S1A). We infer that WT segregants with *mat1* Plus background (genotype II) also gave ura^+^ phenotype, unlike the WT parent, which gives spo^−^, ura^−^ phenotype (Supplementary Figure S1A). Lastly, the spo^+^ phenotype was observed among 47% and 34% segregants among the ts^−^ and ts^+^ segregants, respectively, while the expected level according to the Mendelian segregation would be 50% and 0%, respectively. These results indicate a Non-Mendelian segregation of the *sng2-1*- engendered silencing phenotypes. Furthermore, these states of derepression (spo^+^ and/or or ura^+^) or repression (spo^−^ and/or or ura^−^) exhibited a high level of, though not complete, stability as these switched to the opposite state at a low rate, ranging from 10^−3^ to 10^−6^/generation (Supplementary Figure S1B). Thus, these states represent metastable states of silencing, as reported by Grewal and Klar (22) although their pattern of inheritance is non-Mendelian (See below).

In contrast, a cross between WT strains with genotype I and II did not yield any tetrads with the spo^+^- ura^+^ phenotype (Supplementary Figure S2A). Furthermore, in a cross of *clr3Δ*:: kan^r^ mutant in genotype I having spo^+^-ura^+^ phenotype with WT genotype II strain, only *clr3Δ*(G418^r^) segregants showed spo^+^-ura^+^ phenotype, while *clr3^+^* (G418^s^) segregants did not (Supplementary Figure S2B). These results indicate a Mendelian pattern of segregation in case of a canonical heterochromatin mutant, which is diametrically different from the propagation and inheritance of the silencing defect produced by the *sng2-1*/*cut4^−^* mutation.

**Figure 4.**
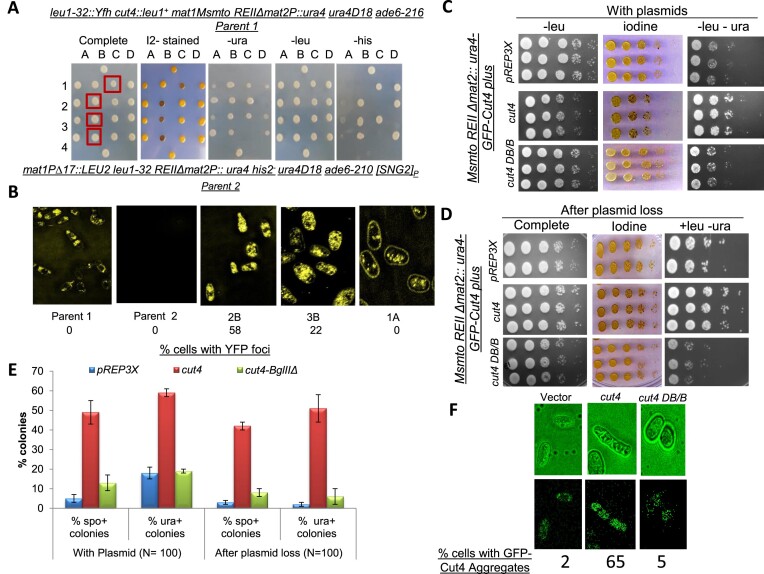
Aggregate formation by fluorescent-tagged Cut4 upon prion conversion (**A**) Tetrads derived from a cross between a WT strain of Minus mating type (genotype I) containing an ectopically integrated YFP-Cut4 (top) and an [SNG2]_P_ strain with genotype II (bottom). Segregants having the putative ‘[SNG2]_M_’ phenotype, are shown in square boxes. (**B**) Confocal microscopic analysis of selected segregants from tetrads analysis shown in (**A**) showing YFP-Cut4 aggregation. (**C**–**F**) *De novo* generation of prion form [SNG2]_M_ upon overexpression of *cut4*. (**C**) Serial dilution spotting assay to show growth on plates lacking uracil and iodine staining phenotype, for transformants of WT strain (*Msmto REIIΔmat2ura4*) containing high copy empty vector (pREP3X), high copy vector containing full length *cut4* gene or an internal deletion *BglIIΔ* (*cut4*DB/B). (**D**) Serial dilution spotting assay showing the persistence of elevated level of ura^+^ phenotype in WT strain after plasmid loss of the transformants shown in (**C**). (**E**) Quantitation of spo^+^ and ura^+^ colonies upon ectopic expression of vector, *cut4* and *cut*4DB/B on high copy vector and upon loss of vectors, as shown in panels (A) and (B), respectively. Data was represented as standard deviation (SD). (**F**) Confocal microscopy pictures of cells of WT strain harboring integrated GFP tagged copy of *cut4* gene upon transformation with different vectors. Numbers denote the percentage of cells containing GFP-Cut4 aggregates.

Prions have been shown to form protein aggregates. To investigate the occurrence of Cut4 as amyloid we repeated the cross shown in Figure 3B except that the one of the parent strains (parent I) was constructed by tagging strain having genotype I (SPA236) with YFH- triple tagged *cut4^+^* gene at the *leu1^−^* locus, which is linked to *mat1*. This strain showed diffused YFP fluorescence (Figure 4B, parent 1 in 4A). As shown earlier in Figure 3B, segregants with varying phenotypes were observed: spo^+^-ura^+^, spo^−^-ura^+^, spo^+^-ura^w^ (where w represents weak phenotype) in the tetrads and non-Mendelian segregation pattern of ura^+^ phenotype was observed (4ura^+^:0 ura^−^, 3ura^+^:1 ura^−^) (Figure 4A). Consistent with the expectation of Cut4 existing as a prion, while parent 1 gave faint fluorescence and the second parent none, the spo^+^-ura^+^ segregants 2B and 3B showed punctuate pattern of YFP fluorescence apparently localized to the cytoplasm (Figure 4B). On the other hand, the segregant 1A with spo^−^- ura^−^ phenotype showed faint fluorescence, like the parent strain with genotype II (parent 1, Figure 4B).

### *De novo* generation of [SNG2]_M_ and Cut4p aggregates upon overexpression of *cut4* and its non-Mendelian inheritance pattern

It has been shown that high level of a prionogenic protein can enhance the rate of its conversion to prion form (11). Accordingly, we transformed the strain SPA236 having genotype I (*leu1^−^ mat1Msmto REIIΔmat2P::ura4*) in which the endogenous copy of the *cut4* gene is tagged with GFP, with empty vector, high copy vector containing *cut4* gene or truncated *cut4* gene carrying deletion of its internal *BglII-BglII* spanning region (*DB/B*), which essentially produces a Cut4 protein truncated after 10 amino acids beyond the *BglII* site. We compared several independent transformants for the iodine staining and *ura4* expression phenotypes. We found that the majority of transformants overexpressing *cut4* gene showed elevated level of spo^+^ (∼50%) and ura^+^ (∼60%) phenotypes (Figure 4C). We further tested whether, as in the case of prions reported earlier, the spo^+^ and ura^+^ phenotype of the transformants persisted after plasmid loss. We subjected three independent colonies (spo^+^ colonies in case of *cut4* overexpression shown in Figure 4C and D) to plasmid loss and repeated the spotting assay. Results showed that the derivatives of spo^+^-ura^+^ continued to exhibit spo^+^-ura^+^ phenotype after loss of the *cut4* plasmid (Figure 4D and E). In contrast, overexpression of *cut4* on a low copy vector had no effect (Supplementary Figure S3A). Generation of prion form upon overexpression of the gene has been shown to be dependent on the presence of a WT copy of the gene (11). However, this experiment could not be performed as *cut4* gene is essential for viability; thus, no negative control like *cut4Δ* is possible. Using a construct allowing addition of an HA tag to the *cut4* or *cut4DB/B* gene, we attempted to confirm that overexpression of the tagged plasmid did elicit high level of Cut4 protein and that of cut4DB/B led to production of a truncated Cut4p. As reported earlier (lane 1 in Figure 5A in Yamashita *et al.*, 1996), we detected a doublet band corresponding to the full length Cut4 (Supplementary Figure S3B, lane 2) and a band of ∼32kD corresponding to truncated Cut4 DB/B (Supplementary Figure S3B, lane 3), confirming the expression of full length as well as the truncated version of Cut4p. Furthermore, the fact that expression of *cut4* and *cut4DB/B* gene on an integrating vector did not abrogate silencing (Supplementary Figure S3A), as also the observation that the derepressed phenotype persisted after the loss of *cut4* gene on high copy vector, as shown above (Figure 4C, D and E), support the inference that the overexpression of Cut4p did abrogate silencing in a heritable manner.

**Figure 5.**
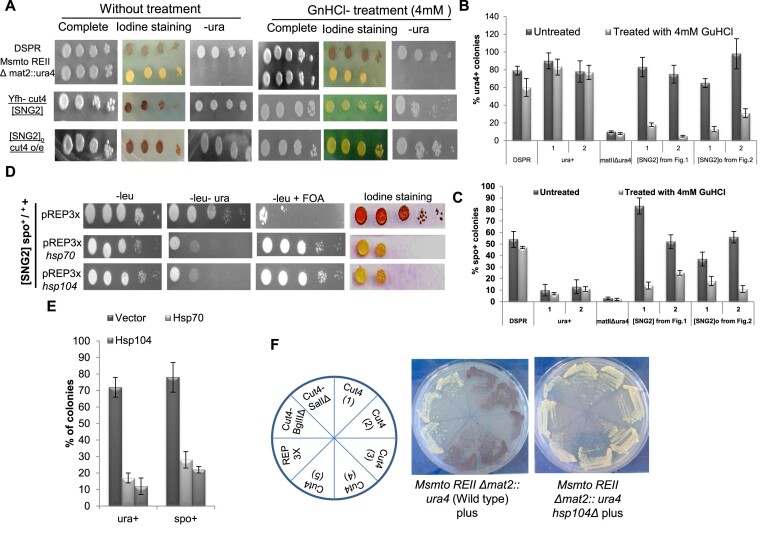
Curing of [SNG2] prion by Gu-HCl and overexpression of heat shock proteins. (**A**) Serial dilution spotting assay of DSPR, *Msmto REIIΔ mat2P::ura4*, SNG2]_M_-YFH cut4 (spo^+^-ura^+^) segregants and [SNG2]^o^ (*cut4* o/e) along with the parental strains on complete plates and plates lacking uracil, with and without treatment of 4 mM Gu-HCl. o/e denotes overexpression. (**B**) Quantitation of percentage of colonie with ura^+^ phenotype on treatment of various strains with 4 mM Gu-HCl. ura^+^ represents control WT, DSPR, SPA236 with genotype *Msmto ura4D18 REIIΔ mat2P::ura4* and two independent [SNG2]_M_ (spo^+^-ura^+^) segregants from Figure 2 and two independent [SNG2]^0^-GFP-cut4 from transformants with ura^+^ phenotype after plasmid loss as shown in Figure 4D. (**C**) Quantitation of percentage of spo^+^ colonies on treatment of different indicated strains with 4 mM Gu-HCl (Iodine staining indicates staining of colonies with iodine after 3 days of growth). (**B**, **C**) Data was represented as SD. (**D**) Curing of prion form of [SNG2]_M_ by overexpression of *hsp* genes in segregant 3B (spo^+^-ura^+^) obtained from cross shown in Figure 2 by serial dilution spotting assay. (**E**) Quantitation of the effect of overexpression of hsp70 and hsp104 on percentage of colonies showing spo^+^ and ura^+^ phenotype. Error bars represent SD. (**F**) Iodine staining of transformants generated from expression of pREP3X, *cut4 DB/B* and *cut4* in WT strain with genotype I (*Msmto REIIΔmat2P::ura4*) and same strain having *hsp104Δ* mutation (*Msmto REIIΔmat2P::ura4 hsp104Δ)*. The transformants were streaked on selective plates (PMA-leu) and colonies stained after 4 days’ growth at 30°C.

To test the mode of inheritance of the spo^+^-ura^+^ phenotype, we first subjected the spo^+^-ura^+^ transformants of *cut4* on high copy vector to plasmid loss. One such spo^+^- ura^+^ derivative obtained after plasmid loss, tentatively designated as [SNG2]^o^, was crossed with the strain SPA302 with genotype II (*mat1PΔ17::LEU2 REIIΔ mat2::ura4*). Tetrad analysis showed that the ura^+^ phenotype segregated in a non-Mendelian pattern with 74% tetrads showing the 4:0, 3:1 and 1:3 segregation pattern (Supplementary Figure S3C).

To detect protein aggregation, we carried out confocal microscopy of the spo^+^-ura^+^ transformants of cells containing GFP-tagged *cut4* gene. Results showed that the transformants obtained with the *cut4* on high copy vector contained aggregates of GFP-Cut4, while those carrying the control vector or truncated *cut4* gene (*DB/B*) did not (Figure 4F). As shown earlier (Figure 4B) the aggregates seem to be localized throughout the cells (Figure 4F). The effect was dependent on expression of full-length gene as the vector containing truncated copy of *cut4* (*DB/B*) had no effect (Figure 4F).

### Curing of [SNG2] by guanidine and dependence on *hsp104*

As protein folding-refolding dynamics underlies the function of proteins, generation of prion form is generally found to be dependent on components of the protein refolding pathway (49,50), although some conflicting results show lack of such dependence (51).

It has been shown that growth in low concentration of guanidine (5 mM) can cure the prion phenotype; Hsp104 is inactivated by low concentration of guanidine, which leads to inhibition of the propagation of prion seeding (11,52,53). To test the effect of guanidine, we first empirically determined the optimum concentration of guanidine. We found that while 5 mM GnHCl reduced cell viability of *S. pombe*, cells grew normally at 4 mM GnHCl (not shown). Therefore, cells of the parental strain SPA236, two independent [SNG2]_M_ segregants from tetrads generated from the cross, as shown in Figure 3B, two independent [SNG2]^o^ derivatives generated by *cut4* overexpression in cells of genotype I, followed by plasmid loss (Figure 4A and D) and two WT ura^+^ colonies were grown in the presence of 4 mM guanidine and effect on spo^+^ and ura^+^ phenotypes was analyzed by plating the treated cells on appropriate medium. The parent strain SPA236 having low level of spo^+^-ura^+^ (5–10% of colonies) exhibited no change in the level of spo^+^-ura^+^ phenotypes on guanidine hydrochloride (Figure 5A–C). Likewise, as controls, the WT ura^+^, h^90^ strains (which sporulate normally and form spo^+^ colonies) showed similarly high levels of ura^+^ phenotype and low percentage of colonies with spo^−^ phenotype before and after the guanidine treatment (Figure 5B and C). Interestingly the [SNG2]_M_ derivatives (Figure 5B and C) showed reduced growth on plates lacking uracil and loss of the spo^+^ phenotype following guanidine treatment. A similar effect was observed in case of [SNG2]° cells (Figure 5A–C), which were derived from [SNG2]_M_ cells generated by overexpression of *cut4* gene, followed by plasmid curing (Figure 4D). However, cells of DSPR showed no effect of guanidine treatment on their spo^+^ and ura^+^ phenotypes (Figure 5A–C).

Overexpression of heat shock proteins Hsp70 and Hsp104 has been shown to cure the prion phenotypes and promote the solubilization of the prion aggregates (21,49–54). Therefore, we transformed the [SNG2]_M_ strain, having spo^+^-ura^+^ phenotype, with empty vector and *hsp104* and *hsp70* genes of *S. pombe* and *S. cerevisiae*, respectively. We monitored the phenotypes of the transformants and the aggregation pattern of prion protein. Consistent with the reported effect of overexpression of *hsp* genes on curing of the prion structure (49), there was a 6–8-fold reduction in the number of transformants having spo^+^-ura^+^ phenotype back to the background level observed in the WT parent strain SPA236 (Figure 5E).

It has been shown that generation and propagation of prions requires presence of molecular chaperones, such as heat shock proteins (55) as they are known to shear the aggregates into small fibrils for transmittance to filial generation. We compared the generation of spo^+^ phenotype by overexpression of *cut4* gene in the WT strain having genotype I (SPA236) to the same strain having *hsp104Δ* mutation. Results show that while all the transformants of the strain SPA236 give spo^+^ phenotype, those having *hsp104Δ* mutation did not (Figure 5F). Thus, the generation of [SNG2]_M_ by overexpression of *cut4* is dependent on *hsp104*.

### Formation of amyloid aggregates and their solubilization by guanidine and Hsp104

**Figure 6.**
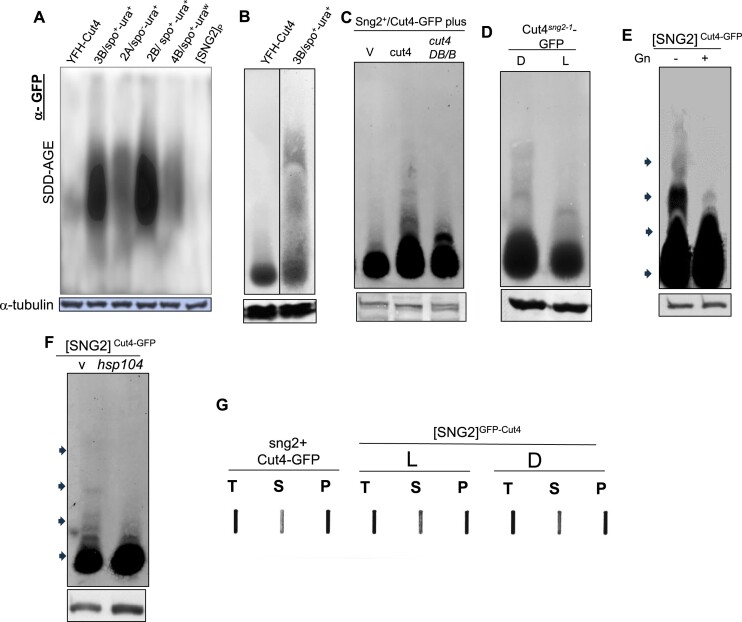
Formation of Cut4 amyloid aggregates in [SNG2] prion form. (**A**) SDD-AGE followed by western blotting with anti-GFP antibody of the protein extracts from segregants shown in Figure 4B. Parent 1: sng2^+^/YFH-Cut4 (lane 1), segregant 3B, with spo^+^-ura^+^ phenotype (lane 2), segregant 2A with phenotype spo^−^-ura^+^ (lane 3), segregant 2B with spo^+^-ura^+^ phenotype (lane 4), segregant 4B with spo^+^-ura^w^ phenotype (lane 5) and Parent 2: [SNG2]_P_ with no YFP-tagged Cut4 (lane 6). (**B**) SDD-AGE under modified conditions showing resolution of the monomeric form of Cut4 from multimeric forms. (**C**) Generation of aggregates of Cut4-GFP upon expression of intact but not truncated Cut4. (**D**) Comparison of the Cut4 prion form in the dark (D) and light (L) derivatives of the *cut4^sng2-1^* mutant. (**E**) Conversion of multimeric forms of Cut4 prion form by growth in presence of guanidine. (**F**) Conversion of amyloid aggregates to monomeric form by overexpression of hsp104. (**C**–**E**) SDD-AGE analysis of Dark and Light derivatives of *sng2-1* mutant having GFP-tagged Cut4^sng2-1^ (**C**), [SNG2] cells grown in the absence and presence of guanidine (**D**) or *hsp104* (**E**). (**A**–**F**) Lower panels represent western blots of samples probed with anti-a-tubulin antibody. Arrowheads represent oligomeric/multimeric bands of Cut4 prion form. (**G**) Fractionation of sng2^+^ and Light (L) and Dark (D) derivatives of [SNG2] having GFP tagged Cut4. Total (T), soluble (S) and pellet (P) fractions were transferred to nitrocellulose membrane by slot blot and blot was probed with anti-GFP antibody.

It has been shown that the prion forms of proteins adopt amyloid structure showing slower electrophoretic mobility under semi-denaturing conditions in agarose gels [semi-denaturing detergent agarose gel electrophoresis (SDD-AGE)]. In this modification, the samples are mixed with 1% SDS but, unlike sodium dodecyl sulphate-polyacrylamide gel electrophoresis (SDS-PAGE), not boiled prior to electrophoresis in the presence of 0.1% SDS (13). Unlike earlier reports, we carried out SDD-AGE in presence of 2% sarkosyl instead of 1% SDS as we failed to observe aggregates under the latter conditions. Results showed that while the parent strain having YFP-tagged Cut4 from the cross shown in Figure 4A showed just a broad band but no aggregates (Figure 6A, lane labelled YFH-Cut4), the segregants 2A, 2B, 3B and 4B, with the phenotypes ura^+^-spo^−^, ura^+^ spo^+^, ura^+^-spo^+^ and ura^w^-spo^+^, respectively, all showed broad band of aggregates (Figure 6A). No band was observed in the untagged parent strain [SNG2]_P_ (Figure 6A). It is interesting that the segregants showing weaker phenotype (Figure 6A, segregants 2A and 4B) have lower intensity of the aggregate band as compared to the segregants with stronger phenotype (Figure 6A, segregants 2B and 3B).

However, we noticed a lack of resolution between the monomeric and oligomeric forms of Cut4 (Figure 6A). After several modifications, we were able to achieve better resolution by reducing the amount of protein by up to ¾ the original amount, a further reduction of sarkosyl concentration in loading buffer to 1% and carrying out blotting at 10°C (Figure 6B). Results showed that while the parent strain contained only the monomeric form, the prion derivatives contain quite distinct monomeric and oligomeric forms (Figure 6B) Accordingly, we performed subsequent experiments under the modified conditions.

We tested for the formation of aggregates in the cells overexpressing *cut4*. Results of SDD-AGE analysis showed that, while oligomeric bands were observed in cells containing GFP-Cut4 overexpressing *cut4*, only a monomeric band was observed in cells transformed with the vector alone or *cut4DB/B* (Figure 6C). We also analyzed the Dark and Light derivatives of the *sng2-1*/*cut4^−^* mutant. The two types of derivatives were tagged with GFP and subjected to SDD-AGE analysis. Results showed the presence of broader spread of oligomeric aggregates in the Dark (D) than the Light derivative, indicating that the expression pattern of the Dark and Light derivatives (Figure 1C and D) correlated with the aggregation behavior of Cut4 (Figure 6D, upper panel).

It has been shown that growth in presence of guanidine can cure the prion by suppressing the expression of hsp104 (11,52,53). We carried out SDD-AGE analysis of cells grown in absence and presence of guanidine. Results showed that growth in presence of guanidine could convert the [SNG2]^GFP-Cut4p^ into monomeric form (Figure 6E). Furthermore, it has been shown that heat shock proteins Hsp70 and Hsp104 can convert the amyloid aggregates into monomers (49). We analyzed two independent ura^+^-spo^+^ transformants obtained by overexpression of *cut4* in strain having GFP-tagged *cut4* gene (Figure 4C and D); these were subjected to loss of the *cut4* plasmid and retransformed with empty vector or *hsp104* gene. A short run of SDD-AGE analysis followed by western blotting with anti-GFP antibody showed the presence of aggregates which spanned the region across the gel up to the sample well in strains transformed with the empty vector (Supplementary Figure S4A, lanes 1 and 2). Interestingly, monomer-sized bands were observed in strain transformed with *hsp104* gene (Supplementary Figure S4A, lanes 3 and 4). SDD-AGE analysis following a longer run, lower amount of protein and blot transfer at 10°C also showed conversion of prionic oligomers of Cut4 into predominantly monomeric form upon expression of hsp104 (Figure 6F). SDS-PAGE analysis followed by western blotting showed equal amount of α-tubulin in all samples (Figure 6A–F, lower panel).

Thus, the apparent discrepancy between the electrophoretic mobility of the aggregate bands in Figure 6B–F and Supplementary Figure S4A can be explained by the shorter duration of the electrophoresis in the latter; the longer duration of electrophoresis in Figure 6E and F could have led to splitting and diffusion of the smaller oligomers of Cut4 through the agarose gel, which did not occur due to the shorter run length of electrophoresis in Supplementary Figure S4A.

Another point of concern is the apparent lower level of oligomeric forms generated de novo in cell overexpressing *cut4* gene, as compared to the prion derivatives obtained from the meiotic cross (compare Figure 6C with Figure 6A and B). This difference could be ascribed to the fact that during *de novo* generation, cells are exposed to higher amounts of Cut4 expressed under the control of the *nmt41* promoter (a moderate strength promoter) for only 3–5 generations in liquid culture, while those of meiotic segregants have grown for over 30–50 generations, first on plates and then in liquid culture, thus giving the latter more time to convert the prionogenic oligomers into multimers. These results also support the idea that small soluble oligomers are quite effective in converting a protein into aggregated form.

It has been shown that upon fractionation by ultracentrifugation, the prion form of Sup35 is fractionated into soluble and pellet fraction and that the one in soluble fraction has the ability of transmission of prion but not the phenotype (46). Therefore, we carried out fractionation of cell extracts of the parent strains and the prionic segregants from Figure 4A into soluble and pellet fractions followed by SDD-AGE and western blotting with anti-GFP antibody. Interestingly and surprisingly, while the pellet fraction in both the parent as well as Light derivative showed the monomeric form of YFP-Cut4, multimeric aggregates were found in the Dark derivatives (Supplementary Figure S4B). Furthermore, while distinct oligomeric forms were detectable in the soluble fraction of dark derivatives these were present at lower level in the parent strain and absent in the Light derivative (arrows, Supplementary Figure S4B). These results suggest a different mode of transmission of [SNG2] from that of Sup35.

Further, to determine whether the [SNG2] has a structurally different form of Cut4p, we subjected the protein extracts to proteinase K digestion, followed by SDS-PAGE and western blotting. Interestingly, we find that while the sng2^+^ and *sng2-1* cells show sensitivity to proteinase K, the [SNG2] strain showed proteinase K resistance, which was lost upon curing with guanidine (Supplementary Figure S4C). These results are consistent with the protease resistant nature of the prion form and its curing by guanidine and hsp104 overexpression, a result also supported by SDD-AGE analysis (Figure 6E and F). These results also indicate a structural difference between the Sng2-1 and [SNG2] form of Cut4p and the restoration of the prion aggregate to normal structure by guanidine.

It is worth noting that the signal in Supplementary Figure S4B for YFP-Cut4 is fainter than those for GFP-Cut4 (Figure 6A). The relatively weaker signal is likely due to lower cross-reactivity of anti-GFP antibody towards YFP as compared to GFP. Thus, the data presented is a balance between the enhancement of signal and minimizing the background.

These results were supported by slot blot analysis, wherein a significant fraction of Cut4-GFP was detectable in the soluble fraction (S) although less than that in the pellet fraction the WT parent strain. In contrast, two different dark derivatives showed a higher level (∼2-fold) amount in the soluble fraction, which was comparable to that in the pellet fraction (Figure 6G).

The apparent greater intensity of aggregates in the prion derivatives as compared to the parent strains is puzzling (Figure Figure 6A), especially in light of the fact that equal intensity bands are observed in SDS-PAGE/western blotting (data not shown). The reason for this is not clear, although similar cases have been reported (75). We speculate that this may be due to amplification of western signal in agarose gel among aggregates of the prion form.

### Dominance and cytoplasmic inheritance of [SNG2]: cytoduction experiment

Because of their cytoplasmic mode of segregation, the prion forms show dominance and cytoplasmic inheritance (9,15,17). This has been demonstrated by doing cytoduction experiment using a karyogamy defective mutant *kar1* in *S. cerevisiae* (3,11). It has been shown that the *tht1Δ* mutant is defective in karyogamy in *S. pombe* (56). The mutant shows lack of fusion of the parental nuclei and meiosis without crossing over. We confirmed this characteristic of *tht1Δ* mutant and observed a lack of intrachromosomal recombination between *leu1* and *his2* markers on *chrII* and parental co-segregation of *chrIII* (*ade6)* and *chrII* marker (*leu1^−^* and *his2^−^*) in a meiotic cross (Supplementary Figure S5).

### Cytoplasmic propagation and dominance of [SNG2} prion form

**Figure 7.**
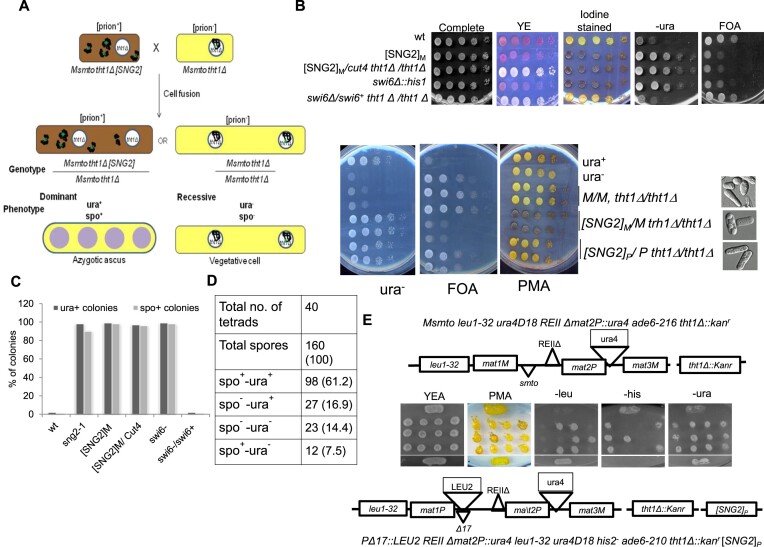
Cytoplasmic inheritance and dominance of [SNG2]. (**A**) Diagrammatic representation of cytoduction experiment for generation of dominant (prion^+^ form) and recessive (prion form) in karyogamy deficient (*tht1Δ*) diploid strain. (**B**) Serial dilution spotting assay of strains with indicated genotypes on complete, YE, PMA^+^, -ura and FOA plates. Colonies growing on PMA^+^ plates were stained with iodine to check sporulation. Lower panel includes the control strains. Pictures on the right side and photomicrographs of the cells subjected to sporulation conditions to induce meiosis. (**C**) Quantitation of ura^+^ and spo^+^ colonies of strains with indicated genotypes after growth on plates lacking uracil and sporulating plates, respectively. (**D**) Tetrads derived from a cross of [SNG2]_P_-*tht1Δ* strain (prion form with genotype II having *tht1Δ*) having spo^−^-ura^+^ phenotype and a WT strain with *tht1Δ* (genotype I) having spo^−^-ura^−^ phenotype, were spotted on YEA and then replica plated to different plates, as indicated. (**E**) Segregation pattern of ura^+^ phenotype among *tht1Δ* mutants in tetrad analysis represented in (**D**).

Stable diploid strains homozygous for *tht1Δ* and genotype I (*leu1^−^ mat1Msmto REIIΔmat2P::ura4*) were constructed by cell fusion and using interallelic complementation of *ade6-210* and *ade6-216* alleles (34) wherein the dominance and cytoplasmic segregation of silencing defect caused by [SNG2] and *swi6Δ* mutation was investigated in heterothallic condition. While a dominant phenotype would manifest as spo^+^ (due to meiosis in the azygotic diploids; see materials and methods) and ura^+^ phenotype, like that in haploid mutant, the spo^−^-ura^−^ minus phenotype would imply recessiveness of the mutation (Figure 7A). Results indicate that the heterozygous diploid [SNG2]_M_/*cut4*^+^ shows spo^+^-ura^+^ phenotype. On the other hand, like the parent diploid strain, the *swi6Δ*/*swi6^+^* diploid shows spo^−^-ura^−^ phenotype, indicating that the *swi6Δ* mutation is recessive (Figure 7B and C). Microscopic examination also revealed the occurrence of azygotic asci in spo^+^ colonies (*tht1Δ, [SNG2]* haploid; *tht1Δ*/ *tht1Δ*, [*SNG2*]/ *cut4^+^* diploid and *tht1Δ swi6Δ* haploid (Figure 7B, lower panel). These results support the cytoplasmic inheritance and dominance of the [SNG2]_M_, while confirming the recessive nature of the *swi6Δ* mutation.

A further test for the cytoplasmic mode of inheritance of [SNG2]_M_ was performed by setting up a cross between a WT *tht1*Δ strain with genotype I and a *tht1*Δ, [SNG2]_P_ strain. Very interestingly, as shown in the normal cross (Figure 3B), several spo^+^-ura^+^ segregants were obtained (Figure 7D and E) with ∼68.7% of segregants being spo^+^ and 78% segregants showed the ura^+^ phenotype (Figure 7D) and non-Mendelian segregation (Figure 7D and E). These results further support the dominance and cytoplasmic basis of inheritance of the [SNG2] prion form.

As another test for cytoplasmic inheritance as well as to test whether the prion form could confer silencing defect at other heterochromatin loci, like *mat3* locus (*mat3::ade6*) and *cen* locus (*otr1R::ade6*), we first crossed cells of [SNG2]_P_ with a naïve WT strain having Minus mating type (*mat1Msmto*) without any reporter gene. Five independent derivatives having *mat1Msmto* locus and no reporter from the above cross, tentatively labeled [SNG2]_M_ 1–5, were then crossed with strains having Plus mating type along with either the *otr1R::ade6* locus (57) [Supplementary Figure S6C (panel b)] or *mat3::ade6* locus (58) [Supplementary Figure S6C (panel a)]. Random spores from these mating as well as the parental strain with the *mat3::ade6* or *otr1R::ade6* locus were streaked on YE plates to assess the effect on silencing of the *ade6* reporter at *otr1R* (Supplementary Figure S6A) *and mat3* (Supplementary Figure S6B) locus, respectively. In both cases, while the parental strains give dark red colonies indicating a transcriptionally silent *ade6* reporter (labeled control, Supplementary Figure S6A and B), the cross involving the derivatives [SNG2]_M_1-5 produced progeny with variable degree of white and pink colonies representing varying levels of derepression of the *ade6* reporter both at *mat3* and *otr1R* loci (Supplementary Figure S6A–C).

To further confirm that the [SNG2] prion can cause silencing defect at the centromere locus, we overexpressed *cut4* gene in the strain having *otr1R::ade6* reporter. A high rate of generation of pink colonies in transformants with high copy vector containing *cut4* gene, but not one containing truncated *cut4 DB/B* gene or the vector control (not shown) in the strain SPA236, as shown earlier, confirms that the putative prion form generated upon overexpression of *cut4* gene can trigger loss of silencing at the centromere locus as well (Supplementary Figure S7).

### Propagation of [SNG2] by protein infection

**Figure 8.**
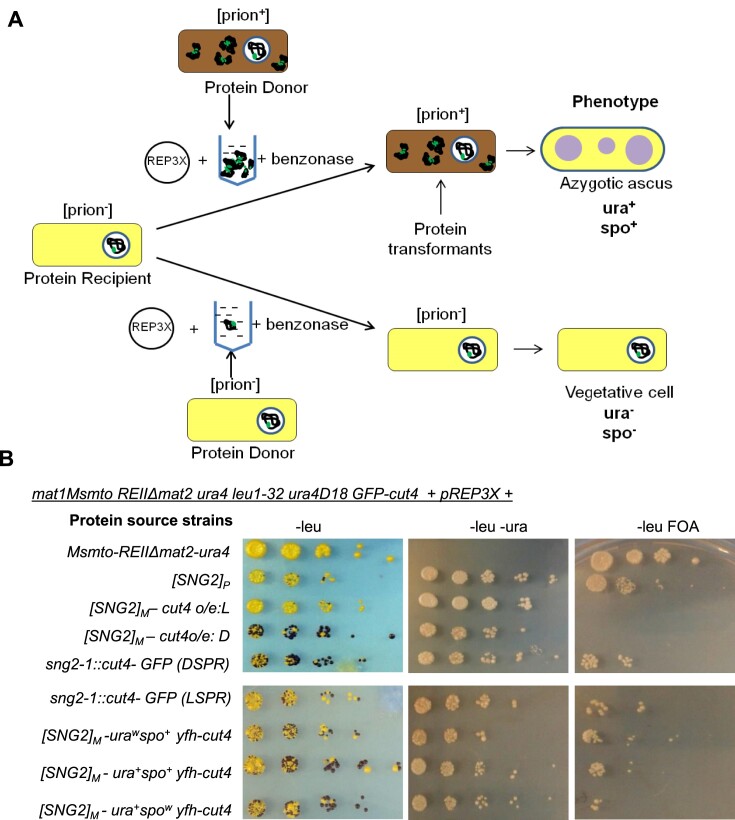
Propagation of [SNG2] by protein transformation. (**A**) Diagrammatic representation of test for infectivity of [SNG2] prion, as described in the ‘Materials and methods' section and the ‘Results’ section. (**B**) Serial dilution spotting assay of wild-type strain (genotype I with GFP tagged cut4) transformed with protein from indicated strains, on complete PMA^+^, -ura and FOA plates. Colonies growing on PMA^+^ plates were stained with iodine to check sporulation.

A key test of protein-based inheritance is the infectivity of protein obtained from the [prion^+^] cells to impart the phenotype upon transformation into the naïve [prion^−^] cells (59). In the present experimental set up, ability of protein extract from [SNG2]_M_ or [SNG2]° cells to induce the phenotype of spo^+^ with haploid meiosis and ura^+^ phenotype when transformed into strain with genotype I (*leu1^−^ mat1Msmto REIIΔmat2P::ura4*) would indicate its infectivity, while inability to do so would indicate it to be non-prion form (schematic representation, Figure 8A). Accordingly, we co-transformed cells of strain SPA236 (genotype I) having GFP-tagged *cut4* gene, with empty vector pREP3 along with benzonase-treated protein extracts prepared from cells of WT, [SNG2]_P_, [SNG2]^o^, DSPR, LSPR and the [SNG2]_M_ segregants obtained from meiotic cross shown in Figure 2. The leu^+^ transformants were screened for the phenotype indicative of silencing. Results show that while cell extracts from WT cells had no effect on expression of the *ura4* reporter and on iodine staining phenotype, extracts of cells of [SNG2]_P_, [SNG2]^o^, [SNG2]_M,_ all caused elevated level of spo^+^- ura^+^ colonies, as indicated by iodine staining, enhanced growth on plates lacking uracil or lack of growth on FOA plates (Figure 8B).

Notably, even extract from cells of DSPR induced the spo^+^-ura^+^ phenotype strongly, while that from cells of LSPR was less effective (Figure 8B). This result suggested that the Dark, spo^+^-ura^+^, derivatives of *sng2-1* mutant with genotype I contained the prion form by itself, while the LSPR may have a lower fraction of prion form as it only exerts a modest effect. The intermediate level of growth on plates lacking uracil and spo^+^ phenotype on protein transformation with LSPR protein extracts mimics the behavior of the parent LSPR strain (Figure 1D). Thus, the two epigenetic states of the *sng2-1* mutant, DSPR and LSPR, initially observed by us (Figure 1) may predominantly represent the strong and weaker prion forms, respectively, of the mutant Cut4p.

### [SNG2] prion form phenocopies *cut4* mutation and affects stress tolerance

**Figure 9.**
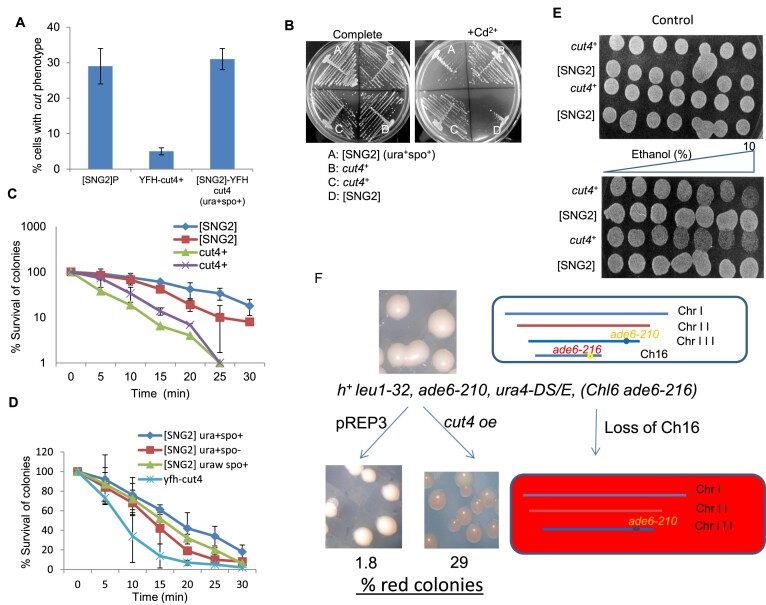
Cells of [SNG2] prion form phenocopy *cut4* mutant and show enhanced stress response. (**A**) Histogram representing the percentage of cells having ‘cut’ phenotype in indicated strains. (**B**) Growth of WT and [SNG2] segregants of a single tetrad on PMA plate containing 20 μM CdCl_2_. (**C**) Plot representing survival of WT and [SNG2] cells subjected to heat stress at 52°C for the indicated time periods. (**D**) Plot representing survival of WT and independent [SNG2] segregants upon exposure to 1 mM H_2_O_2_ for the indicated time periods. (**E**) Spotting assay of cells of two WT and [SNG2] strains each on control plate (top panel) and plate containing 0–10% gradient of ethanol (lower panel). (**F**) Enhanced rate of chromosome loss in [SNG2] cells. The schematic depicts the basis of screen for chromosome loss. Colonies of mutant showing enhanced loss of the artificial chromosome (Ch16 ade6-216) appeared as red colonies, while normal WT cells with high level of chromosome stability produced white colonies on the adenine-limiting YE plate (74).

In general, the prion forms are associated with loss of function of the WT protein function. As *cut4-533* mutant has been associated with ‘cut’ (chromosomes untimely torn) phenotype and enhanced cadmium sensitivity, we checked whether the cells of the [SNG2] prion form may exhibit similar phenotypes. Indeed, we find that cells of both [SNG2]_P_ and [SNG2]_M_ exhibit enhanced ‘cut’ phenotype (Figure 9A) as well as sensitivity to Cd^2+^ (Figure 9B) as compared to the WT parent and closely phenocopies the *cut4-533* mutant (29). In addition, they also show enhanced resistance to thermal, oxidative and ethanol stresses (Figure 9C–E). Furthermore, like the heterochromatin mutant *swi6^−^*, the [SNG2] cells also show enhanced rate of chromosome loss (Figure 9F). Thus, the prion form [SNG2] mimics a functionally defective APC and heterochromatin mutant.

## Discussion

Following up on the discovery of the copper transport associated protein Ctr4 as the first prion in *S. pombe* (60), here we report [SNG2] as a prion form of Cut4 protein from *S. pombe*, which abrogates heterochromatin silencing and affects stress tolerance similar to the *sng2-1*/*cut4* mutant. Sideri *et al.* (60) carried out a predictive analysis and provided a list of probable prion protein (on the basis of Q/N rich prion domains), which does not include Cut4p as a probable prion protein. However, prions [Het-s] from Podospora and PrP^Sc^ from human also lack Q/N rich sequences but are rich in highly charged residues. Likewise, Cut4p is also lacking in Q and N residues but is rich in β-strands (not shown).

Nonetheless, we have shown that the *sng2-1* mutant of *cut4* is a variant that efficiently produces the SNG2] prion. We have also demonstrated that distinct variants of [SNG2] prion exhibit varying phenotypes and aggregation levels of Cut4p. The main significance of this study is that in addition to the chromosomal basis of inheritance, silencing and heterochromatin structure can also be regulated cytoplasmically in a prion-like manner by [SNG2], the prion form of Cut4p, in *S. pombe*.

The findings in the present study originate from the isolation of *sng2-1* a ts mutant allele of *cut4*, which exhibited two states of silencing: the derepressed (DSPR) state, having infectious aggregates of [SNG2] and repressed state (LSPR), with less aggregated and less infectious Cut4^sng2-1^ protein state. The two variants DSPR and LSPR show distinct phenotypes; spo^+^-ura^+^ and spo^−^-ura^w^, respectively, representing strong sporulation (spo^+^) and *ura4* expression (ura^+^), and lack of sporulation (spo^−^) and weak ura expression (ura^w^), as reflected in the level of iodine staining and growth on medium lacking uracil. The dark spo^+^-ura^+^ state denoted here as DSPR, displayed dominance, as it could only be partially complemented by the *cut4* gene on integrating vector but not on high copy vector. Importantly, the spo^+^-ura^+^ phenotype persisted even after the ts^−^ phenotype was segregated out suggesting that it may have generated a self-propagating prion state. A similar scenario occurs upon overexpression of *cut4* gene on a high copy plasmid.

Thus, the DSPR state probably represents a prion form of the mutant protein, which converts the normal Cut4 into prion form. The purified derivatives obtained by crossing and having spo^+^-ura^+^ phenotype were found to fulfill the following prion-like characteristics: (i) non-Mendelian inheritance, (ii) dominance and cytoplasmic inheritance, (iii) curing by guanidine and overexpression of *hsp70* and *hsp104* genes and also propagation of prion form being dependent on *hsp104*, (iv) generation of prion form by overexpression of *cut4* gene, (v) aggregate formation by the prion form of Cut4, and (vi) regeneration of the phenotype by protein transformation. These results lend support to the role of Cut4 as a prion.

Earlier studies in fission yeast showed that heterochromatin structure is regulated in *cis*. For example, Grewal and Klar showed that strain carrying a deletion of the region spanning the *mat2*-*mat3* interval exhibited two alternative states of chromatin that exhibit repressed and derepressed phenotypes (22). Most interestingly, these states were inherited as Mendelian alleles. Furthermore, the role of RNAi at the level of establishment of the repressive epigenetic mark of H3-Lys9 is also shown to be chromosome borne (25). Parallel to RNAi, the histone deacetylase Clr3 is recruited by the ATF/CREB family of proteins and, thereafter, extends in *cis* along with Swi6/HP1 to establish heterochromatin (61).

Earlier work from this lab showed that APC subunits Cut4 and Cut9 interact with Swi6 and the APC and Swi6 act in a mutually cooperative manner to establish heterochromatin structure at centromere and mating type loci (30). The present study shows that a prion form of Cut4 can lead to loss of silencing. It may be speculated that lack of interaction of the prion form of Cut4 with Swi6 may lead to delocalization of Swi6 from heterochromatin and loss of function and thus cause loss of silencing. It is also likely that [SNG2], the prion form of Cut4, is defective in the assembly of APC, as the [SNG2] cells closely phenocopy the *cut4-533* and *sng2-1* mutants. Further investigation will be needed to test these possibilities.

In this regard, it would be interesting to address the mechanism of silencing defect caused by the prion form of Cut4. We carried out ChIP assay to measure the localization of Swi6 and H3-K9-Me2 at the mating type locus. We find that the [SNG2] strain shows a 3-fold reduction, while *swi6Δ* strain shows a 5-fold reduction in localization of Swi6 as compared to wild type (Supplementary Figure S8A and B). A lesser extent of reduction of H3-K9-Me2 was observed (Supplementary Figure S8A and C). These results are consistent with interference with heterochromatin localization of Swi6 and/or Clr4 by the prion form of Cut4.

It is a moot question how the Swi6 delocalization occurs in the prion form. It has been shown that Cut4 interacts with Swi6 and Clr4 *in vivo* and *in vitro* and facilitates their localization to heterochromatin *in vivo*. We speculate that this may be due to lack of interaction between the prion form of Cut4 and Swi6 and/or Clr4. This possibility will be investigated in future studies.

It is pertinent here to mention a recent report showing the role of [ESI1] (**e**xpressed **s**ub-telomeric **i**nformation), a prion form of the Set3 histone deacetylase scaffold Snt1, in generation and transgenerational inheritance of a derepressed chromatin structure at sub-telomeric regions and mating-type locus in *S. cerevisiae* (76). Interestingly, [ESI] interferes the RAP1-mediated silencing (76).

### Variability of [SNG2] prion phenotypes

It was curious to observe variation in phenotypes of the [SNG2] derivatives. For example, at least three types of phenotypes were observed among the meiotic progeny of the cross of [SNG2]_M_ with SPA302 (genotype II), or [SNG2]_P_ with SPA236 (genotype I): spo^+^-ura^+^, spo^+^-ura^w^ and spo^−^-ura^+^. The spo^+^-ura^+^ phenotype represents derepression of both *mat2Pc* and the *mat2P*-linked *ura4* reporter. On the other hand, spo^+^- ura^w^ represents derepression of *mat2Pc* but weaker derepression of the *ura4* reporter, while spo^−^-ura^+^ represents the derepression of *ura4* reporter but not *mat2Pc*. Such diversity in prion phenotypes has been observed in other prions (15,62) and in case of [SNG2], it may represent varying extent of heterochromatin assembly defect. As heterochromatin assembly involves establishment followed by expansion through components like *clr3*, *clr4*, *swi6* etc., the varying phenotypes may represent different extent of expansion of heterochromatin region. Heterochromatin assembly at mating-type region involves action of at least four pathways: (i) *mat2*-linked (31,33) (ii) RNAi-mediated action at *cenH* region (28,63), (iii) ATF-CREB pathway acting at *mat3* (64,65) and (iv) Pap1-dependent pathway (66). Thus, the variability of [SNG2] effect at the *mat3*-linked *ade6* reporter could imply different degrees of effect on expansion of heterochromatin across the *mat2–mat3* interval. Furthermore, the [SNG2] also acts in a similar manner to derepress the *cen::ade6* and *mat3::ade6* reporters, and thereby the heterochromatin structure at the *cen1* and *mat3*, respectively. Interestingly, we find that the specific expression states (eg. spo^+^-ura^+^, spo^+^-ura^w^, spo^−^-ura^+^) can be stably inherited in genetic crosses indicating different stable and heritable form of the [SNG2] prion with distinct structure and function (data not shown). However, the transmission efficiency of the spo^+^-ura^+^ phenotypes during meiotic cross ranged from 62–86% and during protein infectivity assay was around 46–71% among independent transformations, although they originate from crosses between closely related strains, ruling out the possibility of yeast viruses mediating the transmission, which is reported to be 100% (67).

It is pertinent to comment about the difference in prionogenic transmission potential of the fractions of the [SNG2] prion from that of Sup35. It was demonstrated by Dulle *et al.* (46) that the supernatant fraction of the WT lacks any protein while the prion forms show a distinct presence of protein not only in the pellet fraction but also in the supernatant fraction. The presence of protein in the soluble fraction was correlated with ability of transmission of phenotype but not generation of prion. In contrast, the only distinguishing feature correlating with non-transmissibility of [SNG2] is the presence of monomer in the pellet fraction of WT and Light prion derivative, and that of transmissibility with the presence of multimers in the Pellet fraction and oligomers in the Supernatant fraction of the Dark derivative (Supplementary Figure S4B). Thus, the mode of transmission of [SNG2] seems to be different from that of Sup35.

To get an idea about structural differences between normal Cut4 and that in different derivatives, we investigated their sensitivity to proteinase K. Results showed that while the GFP-tagged Sng2^+^ and Sng-2-1 proteins were highly susceptible to proteinase K, GFP-[SNG2] was completely resistant (Supplementary Figure S4C, arrow). Interestingly, growth in presence of guanidine rendered the GFP-[SNG2] protein completely susceptible to proteinase K (Supplementary Figure S4C). These results support other data indicating that [SNG2] has a different structure from that of Sng2^+^p and that it is cured by growth in presence of guanidine.

It is pertinent to address the apparent difference in the intensity of signal of YFP Cut4 in segregant 2B and 3B as compared to parent I (Figure 4B). In fact, this discrepancy is also observed in the SDD-AGE results like those shown in Figure 6A–D but the input blots of SDS-PAGE show closely similar amount in all samples. In this regard, we note similar observation in the case of MLKL (75) and Sup35 (46). This may be due to the possibility that the prion aggregates show better reactivity with or trap the bivalent antibodies in Western blot and may facilitate energy transfer in confocal microscopy experiment.

### Correlation between prion form and the curing of the stress tolerance

A critical test of the role of prion form in stress tolerance is whether the curing of the enhanced stress tolerance can be reversed by subjecting the prionic cells to conditions that cure the effect of prion, i.e. growth in presence of guanidine and overexpression of *hsp104* and *hsp70* genes. However, testing this possibility proved challenging. We tested whether growth in presence of guanidine could reverse the enhanced ethanol tolerance of the prionic cells. Using an assay where a gradient of 0–7% ethanol is established on plates, we confirmed our results of enhanced growth of [SNG2] prion as compared to WT strain after 3 and 4 days of growth (Supplementary Figure S9A). After 5 days, both strains achieved the same colony count although the colony size of the prion cells was larger than that of WT (Supplementary Figure S9A). However, the colony count was not significantly affected by growing WT and prion cells in presence of guanidine (Supplementary Figure S9A).

Next, we measured the growth rate of WT and prion cells in absence and presence of guanidine and 0, 2% and 3% ethanol. Here, we did observe that while the growth curve was both strains was similar in absence and presence of guanidine in normal media (Supplementary Figure S9B, left panel), media containing 2% ethanol (Supplementary Figure S9B, middle panel) and 3% ethanol (Supplementary Figure S9B, right panel) while prion cells showed faster growth in presence of 3% ethanol in absence of guanidine, they exhibited a slower growth rate approaching that of WT cells in presence of guanidine (Supplementary Figure S9B, right panel). These results support the possibility that prion form of Cut4 imparts enhanced stress tolerance. Studying the effect of *hsp* genes in this regard proved somewhat tricky, possibly because expression under the control of the thiamine-regulated promoter nmt1 reaches maximum level after 18–21 h of induction and declines, thereafter, rendering a plate assay difficult. Needless to say, a more rigorous regime needs to be standardized to formally check the role of prion form of Cut4 in stress tolerance.

### Presence of disordered sequences in amyloidogenic region of Cut4p

The inheritance of prion proteins requires both the generation and propagation of the amyloidogenic aggregates. Many of proteins form aggregates but they do not propagate in a non-Mendelian fashion to their filial generations. These include inclusion bodies, folding aggregates and amorphous bodies. Another class of proteins, the intrinsically disordered proteins can also form aggregates, which propagate in a non-Mendelian fashion to their daughter cells: their propagation is dependent on molecular chaperone and some of these can form amyloids (21,68).

As the [SNG2] derivatives obtained either by genetic cross showed aggregates of Cut4p that are resistant to 2% sarkosyl, we investigated the presence of aggregate-forming and amyloidogenic domains in Cut4p, utilizing bioinformatics tools. As most of the prion prediction algorithms are based on the detection of Q/N rich regions, the proper prion scans could not be generated as, like human Prp and fungal HET-s prions, Cut4p lacks regions rich in glutamine and asparagine (69). Using the PSI-PRED and Disopred 3 softwares (47) on the Protein Structure Prediction Server PSIPRED of the UCL Department of Computer Science: Bioinformatics Group, we predicted the features of secondary structure (Supplementary Figure S10A) and regions having potential to form Intrinsically disordered domains (Supplementary Figure S10B). We find a number of regions in Cut4 having the potential to form Intrinsically disordered domain, the notable being residues 195–210, 418–457, 520–529, 551–578, etc. Of particular interest is the region 195–210, which is surrounded by long stretches of β-strand (Supplementary Figure S10A and B). In future studies, we will investigate the role of the domains identified by the DISOPRED3 software in inducing prions by doing domain swapping experiments with SUP35.

Curiously, the *sng2-1* mutation at residue 984 does not map to any one of the regions predicted above. We suggest that the *sng2-1* mutation may accentuate the potential of the putative prionogenic regions to form prions, as has been shown earlier (70). Furthermore, it is interesting that the *sng2-1* mutation (amino acid residue 984) is located in the region spanned by the *Bgl*II sites, whose deletion abolishes the prionogenic potential of Cut4, as shown in Figure 4.

### Cellular consequences of prion form of Cut4

It is a moot question whether the aggregated prion form of Cut4 performs its normal physiological functions. We find that the [SNG2] cells display similar sensitivity to heavy metal ions like, Cd^2+^, as the *sng2-1* and *cut4-533* mutants (29,71). Thus, the prion form of Cut4 phenocopies the *cut4* mutant, representing a loss of function, as expected in case of prions. Likewise, similar to the *cut4* and other *‘cut’* mutants, the [SNG2] cells also show high incidence of ‘*cut*’ (chromosomes untimely torn) phenotype, which represents deregulation of cell cycle progression (29,71). Furthermore, like *sng2-1* and *swi6* mutants, [SNG2] cells also show enhanced rate of chromosome loss indicating that the prion form of Cut4 causes chromosome instability, like silencing mutant *swi6* (30).

### Evolutionary significance

It has been proposed that some prions confer advantage on cells to survive and adapt to various stresses (72). In agreement, we find that cells of the [SNG2] prion exhibited enhanced tolerance to various environmental stresses, like ethanol stress, thermal stress and oxidative stress. It is puzzling that Cut4, a protein performing an important and evolutionarily conserved role of regulating cell cycle progression in all eukaryotes, has evolved to retain regions that makes it potentially non-functional and able to impart stress tolerance. Computational analyses reveal that regions having potential to form amyloids are conserved in Cut4 sequences in other species as well (not shown), thus supporting the notion that such sequences have been selected during evolution and may provide selective advantage in response to environmental changes.

Paradoxically, the [SNG2] cells also exhibited cell cycle defect and enhanced chromosomal loss, conditions characteristic of disease states like cancer. It is possible that cell cycle defects and chromosomal defects may be instrumental in conferring enhanced stress tolerance, a trait exhibited by cancer cells, which remains to be investigated further. The enhanced stress tolerance of cells bearing [SNG2] suggests that the [SNG2] prion may facilitate the adaptation of the *S. pombe* cells to various environmental changes, thus providing a basis for selection of their propagation during mitosis and meiosis.

That Cut4^sng2-1^ mutant protein may adopt a metastable prion structure more efficiently than WT Cut4p is not unprecedented. For example [PSI^+^] has been shown to arise 5000-fold more frequently in the Sup35p mutant containing two additional oligopeptide repeats (73). Likewise, H2p mutant of Ure2p causes 1000-fold increase in the rate of [URE3] induction although this and other similar mutations lie outside the prion domain (70). It has been suggested that conformational flexibility may enhance the frequency of prion induction.

Another pertinent point is the partial overlap between the monomer and the aggregate form of Cut4 in the prion derivatives. Only in a short electrophoretic runs some distinction can be observed, although there is still a small overlap between the leading edge of the prion aggregates and the trailing edge of the prion sample cured by *hsp104* (Supplementary Figure S4A). We think that this has to viewed in light of the fact that Cut4 is an essential gene and performs important functions during cell cycle as a component of APC: metaphase-to-anaphase transition and cell cycle exit. Thus, given that the aggregated form of Cut4 would negatively impact its function, some functionally active fraction has to be present to perform the essential function, as argued earlier (77).

In conclusion, this is the first report of a prion affecting heterochromatin structure in eukaryotes. The ease of doing genetics, biochemistry and cell biology in fission yeast should facilitate a deeper insight of molecular mechanisms and significance of Cut4p prion both in yeast in future studies.

## Supplementary Material

gkae1136_Supplemental_File

## Data Availability

The data underlying this article are available in the article and in its online supplementary material.

## Notes

Present address: Suman Sharma, Amity University Punjab, GMADA It City, Block A St, JLPL Industrial Area, Sahibzada Ajit Singh Nagar, Punjab 140306, India

Present address: Suchita Srivastava, Central Research Institute, Kasauli, Distt, Solan, Himachal Pradesh 173204, India

Present address: Rudra Narayan Dubey, Teva North America – US Headquarters, 400 Interpace Parkway, #3 Parsippany, NJ 07054, USA

Present address: Poonam Mishra, Krishna School of Science, KPG University, Vadodara, Gujarat 390002, India

Present address: Jagmohan Singh, Amity University Punjab, GMADA It City, Block A St, JLPL Industrial Area, Sahibzada Ajit Singh Nagar, Punjab 140306, India

## References

[1] Prusiner S.B. Novel proteinaceous infectious particles cause scrapie. Science. 1982; 216:136–144. 6801762 10.1126/science.6801762

[2] Oesch B. , Westaway D., Wälchli M., McKinley M.P., Kent S.B., Aebersold R., Barry R.A., Tempst P., Teplow D.B., Hood L.E. A cellular gene encodes scrapie PrP 27-30 protein. Cell. 1985; 40:735–746. 2859120 10.1016/0092-8674(85)90333-2

[3] Moore R.C. , Hope J., McBride P.A., McConnell I., Selfridge J., Melton D.W., Manson J.C. Mice with gene targeted prion protein alterations show that Prnp, Sinc and Prni are congruent. Nat. Genet. 1998; 18:118–125. 9462739 10.1038/ng0298-118

[4] Prusiner S.B. Prions. Proc. Natl Acad. Sci. U.S.A. 1998; 95:13363–13383. 9811807 10.1073/pnas.95.23.13363 PMC33918

[5] Zabel M.D. , Reid C. A brief history of prions. FEMS Path Disease. 2015; 73:ftv087. 10.1093/femspd/ftv087 PMC4626585 26449713

[6] Alper T. , Cramp W.A., Haig D.A., Clarke M.C. Does the agent of scrapie replicate without nucleic acid?. Nature. 1967; 214:764–766. 4963878 10.1038/214764a0

[7] Griffith J.S. Self replication and Scrapie. Nature. 1967; 215:1043–1044. 4964084 10.1038/2151043a0

[8] Lacroute F. Non-Mendelian mutation allowing ureidosuccinic acid uptake in yeast. J. Bacteriol. 1971; 106:519–522. 5573734 10.1128/jb.106.2.519-522.1971 PMC285125

[9] Aigle M. , Lacroute F. Genetical aspects of [URE3], a non-mitochondrial, cytoplasmically inherited mutation in yeast. Mol. Gen. Genet. 1975; 136:327–335. 16095000 10.1007/BF00341717

[10] Cox B. PSI], a cytoplasmic suppressor of super-suppression in yeast. Heredity. 1965; 20:505–521.

[11] Wickner R.B. URE3] as an altered URE2 protein: evidence for a prion analog in *Saccharomyces cerevisiae*. Science. 1994; 264:566–569. 7909170 10.1126/science.7909170

[12] Edskes H.K. , Gray V.T., Wickner R.B. The [URE3] prion is an aggregated form of Ure2p that can be cured by overexpression of Ure2p fragments. Proc. Natl Acad. Sci. U.S.A. 1999; 96:1498–1503. 9990052 10.1073/pnas.96.4.1498 PMC15494

[13] Alberti S. , Halfmann R., Lindquist S. Biochemical, cell biological and genetic assays to analyze amyloid and prion aggregation in yeast. Methods Enzymol. 2010; 470:709–734. 20946833 10.1016/S0076-6879(10)70030-6

[14] Wickner R.B. , Masison D.C., Edskes H.K. [PSI] and [URE3] as yeast prions. Yeast. 1995; 11:1671–1685. 8720070 10.1002/yea.320111609

[15] Derkatch I.L. , Chernoff Y.O., Kushnirov V.V., Inge-Vechtomov S.G., Liebman S.W. Genesis and variability of [PSI] prion factors in *Saccharomyces cerevisiae*. Genetics. 1996; 144:1375–1386. 8978027 10.1093/genetics/144.4.1375 PMC1207691

[16] Derkatch I.L. , Bradley M.E., Hong J.Y., Liebman S.W. Prions affect the appearance of other prions: the story of [PIN(+)]. Cell. 2001; 106:171–178. 11511345 10.1016/s0092-8674(01)00427-5

[17] Sondheimer N. , Lindquist S. Rnq1: an epigenetic modifier of protein function in yeast. Mol. Cell. 2000; 5:163–172. 10678178 10.1016/s1097-2765(00)80412-8

[18] Patel B.K. , Gavin-Smyth J., Liebman S.W. The yeast global transcriptional co-repressor protein Cyc8 can propagate as a prion. Nat. Cell Biol. 2009; 11:344–349. 19219034 10.1038/ncb1843 PMC2667906

[19] Du Z. , Park K.W., Yu H., Fan Q., Li L. Newly identified prion linked to the chromatin- remodeling factor Swi1 in *Saccharomyces cerevisiae*. Nat. Genet. 2008; 40:460–465. 18362884 10.1038/ng.112 PMC2633598

[20] Coustou V. , Deleu C., Saupe S., Begueret J. The protein product of the het-s heterokaryon in compatibility gene of the fungus *Podospora anserina* behaves as a prion analog. Proc. Natl Acad. Sci. U.S.A. 1997; 94:9773–9778. 9275200 10.1073/pnas.94.18.9773 PMC23266

[21] Uptain S.M. , Lindquist S. Prions as protein-based genetic elements. Ann. Rev. Microbiol. 2002; 256:703–741. 10.1146/annurev.micro.56.013002.100603 12142498

[22] Grewal S.I. , Klar A.J. Chromosomal inheritance of epigenetic states in fission yeast during mitosis and meiosis. Cell. 1996; 86:95–101. 8689692 10.1016/s0092-8674(00)80080-x

[23] Nakayama J. , Rice J.C., Strahl B.D., Allis C.D., Grewal S.I. Role of histone H3 lysine 9 methylation in epigenetic control of heterochromatin assembly. Science. 2001; 292:110–113. 11283354 10.1126/science.1060118

[24] Bannister A.J. , Zegerman P., Partridge J.F., Miska E.A., Thomas J.O., Allshire R.C., Kouzarides T. Selective recognition of methylated lysine 9 on histone H3 by the HP1 chromo domain. Nature. 2001; 410:120–124. 11242054 10.1038/35065138

[25] Hall I.M. , Shankaranarayana G.D., Noma K., Ayoub N., Cohen A., Grewal S.I. Establishment and maintenance of a heterochromatin domain. Science. 2002; 297:2232–2237. 12215653 10.1126/science.1076466

[26] Haldar S. , Saini A., Nanda J.S., Saini S., Singh J. Role of Swi6/HP1 self-association- mediated recruitment of Clr4/Suv39 in establishment and maintenance of heterochromatin in fission yeast. J. Biol. Chem. 2011; 286:9308–9320. 21224386 10.1074/jbc.M110.143198 PMC3058978

[27] Canzio D. , Liao M., Naber N., Pate E., Larson A., Wu S., Marina D.B., Garcia J.F., Madhani H.D., Cooke R. et al. A conformational switch in HP1 releases auto-inhibition to drive heterochromatin assembly. Nature. 2013; 496:377–381. 23485968 10.1038/nature12032 PMC3907283

[28] Volpe T.A. , Kidner C., Hall I.M., Teng G., Grewal S.I., Martienssen R.A. Regulation of heterochromatic silencing and histone H3 lysine-9 methylation by RNAi. Science. 2002; 297:1833–1837. 12193640 10.1126/science.1074973

[29] Yamashita Y.M. , Nakaseko Y., Samejima I., Kumada K., Yamada H., Michaelson D., Yanagida M. 20S cyclosome complex formation and proteolytic activity inhibited by the cAMP/PKA pathway. Nature. 1996; 384:276–279. 8918880 10.1038/384276a0

[30] Dubey R.N. , Nakwal N., Bisht K.K., Saini A., Haldar S., Singh J. Interaction of APC/C-E3 Ligase with Swi6/HP1 and Clr4/Suv39 in heterochromatin assembly in fission yeast. J. Biol. Chem. 2009; 284:7165–7176. 19117951 10.1074/jbc.M806461200 PMC2652303

[31] Thon G. , Klar A.J. The clr1 locus regulates the expression of the cryptic mating-type loci of fission yeast. Genetics. 1992; 131:287–296. 1644273 10.1093/genetics/131.2.287 PMC1205004

[32] Ekwall K. , Ruusala T. Mutations in rik1, clr2, clr3 and clr4 genes asymmetrically derepress the silent mating-type loci in fission yeast. Genetics. 1994; 136:53–64. 8138176 10.1093/genetics/136.1.53 PMC1205792

[33] Thon G. , Cohen A., Klar A.J. Three additional linkage groups that repress transcription and meiotic recombination in the mating-type region of *Schizosaccharomyces pombe*. Genetics. 1994; 138:29–38. 8001791 10.1093/genetics/138.1.29 PMC1206135

[34] Moreno S. , Klar A., Nurse P. Molecular genetic analysis of fission yeast *Schizosaccharomyces pombe*. Methods Enzymol. 1991; 194:795–823. 2005825 10.1016/0076-6879(91)94059-l

[35] Bahler J. , Wu J.Q., Longtine M.S., Shah N.G., McKenzie A., Steever A.B., Wach A., Philippsen P., Pringle J.R. Heterologous modules for efficient and versatile PCR-based gene targeting in *Schizosaccharomyces pombe*. Yeast. 1998; 14:943–951. 9717240 10.1002/(SICI)1097-0061(199807)14:10<943::AID-YEA292>3.0.CO;2-Y

[36] Matsuyama A. , Arai R., Yashiroda Y., Shirai A., Kamata A., Sekido S., Kobayashi Y., Hashimoto A., Hamamoto M., Hiraoka Y. et al. ORFeome cloning and global analysis of protein localization in the fission yeast *Schizosaccharomyces pombe*. Nat. Biotechnol. 2006; 24:841–847. 16823372 10.1038/nbt1222

[37] Sipiczki M. , Ferenczy L. Protoplast fusion of *Schizosaccharomyces pombe* auxotrophic mutants of identical mating-type. Mol. Gen. Genet. 1977; 151:77–81. 865481 10.1007/BF00446915

[38] Kipling D. , Kearsey S.E. Reversion of autonomously replicating sequence mutations in *S. cerevisiae*. Creation of eukaryotic replication origin within a prokaryotic vector-DNA. Mol. Cell Biol. 1990; 10:265–272. 2403637 10.1128/mcb.10.1.265-272.1990 PMC360734

[39] Eaglestone S.S. , Cox B.S., Tuite M.F. Translation termination efficiency can be regulated in *Saccharomyces cerevisiae* by environmental stress through a prion-mediated mechanism. EMBO J. 1999; 18:1974–1981. 10202160 10.1093/emboj/18.7.1974 PMC1171282

[40] Máté G. , Gazdag Z., Mike N., Papp G., Pócsi I., Pesti M. Regulation of oxidative stress- induced cytotoxic processes of citrinin in the fission yeast *Schizosaccharomyces pombe*. Toxicon. 2014; 90:155–166. 25128706 10.1016/j.toxicon.2014.08.005

[41] Gasset-Rosa F. , Coquet A.-S., Moreno-del Alamo M., Chen P., Song X., Serrano A.M., Fernandez-Tresquerres M.E., dela Espina S.M.-D., Lindner A.B., Giraldo R Direct assessment in bacteria of prinoid propagation and phenotype selection by Hsp70 chaperone. Mol. Micro. 2014; 91:1070–1087. 10.1111/mmi.12518 24417419

[42] Kryndushkin D.S. , Alexandrov I.M., Ter-Avanesyan M.D., Kushnirov V.V. Yeast [PSI] prion aggregates are formed by small Sup35 polymers fragmented by Hsp104. J. Biol. Chem. 2003; 278:49636–49643. 14507919 10.1074/jbc.M307996200

[43] Beach D. , Nurse P. High-frequency transformation of the fission yeast *Schizosaccharomyces pombe*. Nature. 1981; 290:140–142. 22442847 10.1038/290140a0

[44] Tanaka M. , Chien P., Naber N., Cooke R., Weisman J. Conformational variations in an infectious protein determine prion strain differences. Nature. 2004; 428:323–328. 15029196 10.1038/nature02392

[45] King C.-Y. , Diaz-Avalos R. Protein-only transmission of three yeast prion strains. Nature. 2004; 428:319–323. 15029195 10.1038/nature02391

[46] Dulle J.E. , Bouttenot R.E., Underwood L.A., True H.L. Soluble oligomers are sufficient for transmission of a yeast prion but do not confer phenotype. J. Cell Biol. 2013; 203:197–204. 24145167 10.1083/jcb.201307040 PMC3812976

[47] Jones D.T. , Cozetto D. DISOPRED 3: precise disordered region predictions with annotated protein binding activity. Bioinformatics. 2015; 31:857–863. 25391399 10.1093/bioinformatics/btu744 PMC4380029

[48] Arcangioli B. , Klar A.J. A novel switch-activating site (SAS1) and its cognate binding factor (SAP1) required for efficient mat1 switching in *Schizosaccharomyces pombe*. EMBO J. 1991; 10:3025–3032. 1915277 10.1002/j.1460-2075.1991.tb07853.x PMC453017

[49] Glover J.R. , Lindquist S. Hsp104, Hsp70, and Hsp40: a novel chaperone system that rescues previously aggregated proteins. Cell. 1998; 94:73–82. 9674429 10.1016/s0092-8674(00)81223-4

[50] Reidy M. , Sharma R., Masison D.C. *Schizosaccharomyces pombe* disaggregation machinery chaperones support *Saccharomyces cerevisiae* growth and prion propagation. Eukaryot. Cell. 2013; 12:739–745. 23504563 10.1128/EC.00301-12 PMC3647771

[51] Sénéchal P. , Arseneault G., Leroux A., Lindquist S., Rokeach L.A. The *Schizosaccharomyces pombe* Hsp104 disaggregase is unable to propagate the [PSI] prion. PLoS One. 2009; 4:e6939. 19759825 10.1371/journal.pone.0006939 PMC2736384

[52] Tuite M.F. , Mundy C.R., Cox B.S. Agents that cause a high frequency of genetic change from [*PSI*+] to [*psi-*] in *Saccharomyces cerevisiae*. Genetics. 1981; 98:691–711. 7037537 10.1093/genetics/98.4.691 PMC1214469

[53] Ferreira P.C. , Ness F., Edward S.R., Cox B.S., Tuite M.F. The elimination of the Yeast [PSI+] prion by guanidine hydrochloride is the result of Hsp104 inactivation. Mol. Microbiol. 2001; 40:1357–1369. 11442834 10.1046/j.1365-2958.2001.02478.x

[54] Wickner R.B. , Shewmaker F.P., Bateman D.A., Edskes H.K., Gorkovskiy A., Dayani Y., Bezsonov E.E. Yeast prions: structure, biology, and prion-handling systems. Microbiol. Mol. Biol. Rev. 2015; 79:1–17. 25631286 10.1128/MMBR.00041-14 PMC4402965

[55] Chernoff Y.O. , Kiktev D. Dual role of ribosome-associated chaperones in prion formation and propagation. Curr. Genet. 2016; 62:677–685. 26968706 10.1007/s00294-016-0586-2

[56] Tange Y. , Horio T., Shimanuki M., Ding D.Q., Hiraoka Y., Niwa O. A novel fission yeast gene, tht1^+^, is required for the fusion of nuclear envelopes during karyogamy. J. Cell Biol. 1998; 140:247–258. 9442101 10.1083/jcb.140.2.247 PMC2132580

[57] Allshire R.C. , Javerzat J.P., Redhead N.J., Cranston G. Position effect variegation at fission yeast centromeres. Cell. 1994; 76:157–169. 8287474 10.1016/0092-8674(94)90180-5

[58] Thon G. , Bjerling K.P., Nielsen I.S. Localization and properties of a silencing element near the mat3M mating-type cassette of *Schizosaccharomyces pombe*. Genetics. 1999; 151:945–963. 10049914 10.1093/genetics/151.3.945 PMC1460531

[59] Sideri T.C. , Stojanovski K., Tuite M.F., Grant C.M. Ribosome-associated peroxiredoxins suppress oxidative stress-induced de novo formation of the [PSI+] prion in yeast. Proc. Natl. Acad Sci. USA. 2010; 107:6394–6399. 20308573 10.1073/pnas.1000347107 PMC2851942

[60] Sideri T. , Yashiroda Y., Ellis D.A., Rodríguez-López M., Yoshida M., Tuite M.F., Bähler J. The copper transport associated protein Ctr4 can form prion-like epigenetic determinants in *Schizosaccharomyces pombe*. Microb. Cell. 2017; 4:16–28. 28191457 10.15698/mic2017.01.552 PMC5302157

[61] Yamada T. , Fischle W., Sugiyama T., Allis C.D., Grewal S.I. The nucleation and maintenance of heterochromatin by a histone deacetylase in fission yeast. Mol. Cell. 2005; 20:173–185. 16246721 10.1016/j.molcel.2005.10.002

[62] True H.L. , Lindquist S. A yeast prion provides a mechanism for genetic variation and phenotypic diversity. Nature. 2000; 407:477–483. 11028992 10.1038/35035005

[63] Grewal S.I. , Klar A.J. A recombinationally repressed region between mat2 and mat3 loci shares homology to centromeric repeats and regulates directionality of mating- type switching in fission yeast. Genetics. 1997; 146:1221–1238. 9258669 10.1093/genetics/146.4.1221 PMC1208070

[64] Jia S. , Yamada T., Grewal S.I. Heterochromatin regulates cell type-specific long-range chromatin interactions essential for directed recombination. Cell. 2004; 119:469–480. 15537537 10.1016/j.cell.2004.10.020

[65] Kim H.S. , Choi E.S., Shin J.A., Jang Y.K., Park S.D. Regulation of Swi6/HP1 dependent heterochromatin assembly by cooperation of components of the mitogen-activated protein kinase pathway and a histone deacetylase Clr6. J. Biol. Chem. 2004; 279:42850–42859. 15292231 10.1074/jbc.M407259200

[66] Kumar A. , Nanda J.S., Saini S., Singh J. An RNAi-independent role of the AP-like stress response factor Pap1 in centromere and mating-type silencing in *Schizosaccaromyces Pombe*. J. Biosc. 2021; 46:74. 34344846

[67] Wesolowski M. , Wickner R.B. Two new double-stranded RNA molecules showing non-Mendelian inheritance and heat inducibility in *Saccharomyces cerevisiae*. Mol. Cell Biol. 1984; 4:181–187. 6366509 10.1128/mcb.4.1.181-187.1984 PMC368673

[68] Chakrabortee S. , Byers J.S., Jones S., Garcia D.M., Bhullar B., Chang A., She R., Lee L., Fremin B., Lindquist S. et al. Intrinsically disordered proteins drive emergence and inheritance of biological traits. Cell. 2016; 167:369–381. 27693355 10.1016/j.cell.2016.09.017 PMC5066306

[69] Espargaró A. , Busquets M.A., Estelrich J., Sabate R. Predicting the aggregation propensity of prion sequences. Virus Res. 2015; 207:127–135. 25747492 10.1016/j.virusres.2015.03.001

[70] Fernandez-Bellot E. , Guillemet E., Cullin C. The yeast prion [URE3] can be greatly induced by a functional mutated URE2 allele. EMBO J. 2000; 19:3215–3222. 10880435 10.1093/emboj/19.13.3215 PMC313950

[71] Yanagida M. , Yamashita Y.M., Tatebe H., Ishii K., Kumada K., Nakaseko Y. Control of metaphase-anaphase progression by proteolysis: cyclosome function regulated by the protein kinase A pathway, ubiquitination and localization. Philos. Trans. R. Soc. Lond. B Biol. Sci. 1999; 354:1559–1569. 10582241 10.1098/rstb.1999.0499 PMC1692673

[72] Suzuki G. , Shimazu N., Tanaka M. A yeast prion, Mod5, promotes acquired drug resistance and cell survival under environmental stress. Science. 2012; 336:355–359. 22517861 10.1126/science.1219491

[73] Liu J.J. , Lindquist S. Oligopeptide-repeat expansions modulate ‘protein-only’ inheritance in yeast. Nature. 1999; 400:573–576. 10448860 10.1038/23048

[74] Ekwall K. , Olsson T., Turner B.M., Cranston G., Allshire R.C. Transient inhibition of histone deacetylation alters the structural and functional imprint at fission yeast centromeres. Cell. 1997; 91:1021–1032. 9428524 10.1016/s0092-8674(00)80492-4

[75] Liu S. , Liu H., Johnston A., Hanna-Addams S., Reynoso E., Xiang Y., Wang Z. MLKL forms disulfide bond-dependent amyloid-like polymerase to induce ncroptosis. Proc. Natl Acad. Sci. U.S.A. 2017; 114:E7450–E7459. 28827318 10.1073/pnas.1707531114 PMC5594682

[76] Harvey Z.H. , Chakravarty A.K., Futia R.A., Jarosz D.F. A prion epigenetic switch establishes active chromatin state. Cell. 2020; 180:928–940. 32109413 10.1016/j.cell.2020.02.014 PMC7195540

[77] Wickner R.B. , Kelly A.C. Prions are affected by evolution at two levels. Cell. Mol. Life Sci. 2016; 73:1131–1144. 26713322 10.1007/s00018-015-2109-6 PMC4762734

