# Supplementary material for: [SNG2], a prion form of Cut4/Apc1, confers non-Mendelian inheritance of heterochromatin silencing defect in fission yeast
Source: Nucleic Acids Res. 2024 Nov 20;52(22):13792–811. doi: 10.1093/nar/gkae1136 (PMC11662930; doi:10.1093/nar/gkae1136)
Supplement: gkae1136_Supplemental_File [file gkae1136_supplemental_file.pdf]

Supplementary Data

High mitotic stability of the [SNG2] prion forms

We determined the stability of the $[SNG2]_M$ prion. We found that three independent [SNG2] segregants from the cross shown in **Figure 2** showed stable though variable phenotypes, with 66-98% and 60-96% of colonies showing spo^+ and ura^+ phenotypes, respectively, over repeated freeze- thawing cycles (not shown). The original forms of the *sng2-1* mutant (DSPR and LSPR) and various [SNG2] derivatives with spo^+-ura^+, spo^--ura^+, spo^+-ura^w and spo^w- ura^+ phenotypes were inherited stably during mitosis, showing a switch to the opposite state at rate/generation ranging from $1.7X\ 10^{-4}$ to $5.6X10^{-5}$/generation for spo^+ phenotype and $1X10^{-4}$ to $9.5X10^{-5}$/generation for ura^+ phenotype, respectively **(Figure S1B)**. Similarly, the [SNG2] prion phenotype was also stably propagated during meiosis: nearly 56% and 23% segregants of a meiotic cross retained the spo^+ and ura^+ phenotypes, respectively, during meiosis. Similar stable pattern of inheritance was observed during repeated crosses.

Table S1. Strains used in the study

**Strain**	**Genotype**
**SPA 236**	*mat1Msmto leu1-32 ura4D18 REII Δmat2P::ura4 ade6-216*
**SPA302**	*mat1PΔ17::leu2 REIIΔmatP2::ura4 leu1-32 ura4D18* $his2^-$ *ade6-210*
**FY566**	h^+ *leu1-32, ade6-210, ura4-DS/E, (Chl6 ade6-216)*
**FY2002**	h^+ *leu1-32 ura4DS/E ade6DN/N imr1L::ura4 otr1R::ade6*
**PG1672**	*mat1PΔ17::LEU2 mat3-M(RV) ::ade6ori 1 leu1-32 ura-D18 ade6-210*
**SPJ1009**	*mat1Msmto leu 1-32 REIIIΔmat3:: ade6 ade6DN/N*
**DSPR**	*mat1Msmto leu1-32 ura4D18 REII Δmat2P::ura4 ade6-216 sng2-1(dark derivative)*
**LSPR**	*mat1Msmto leu1-32 ura4D18 REII Δmat2P::ura4 ade6-216 sng2-1 (lightderivative)*
**SPJ25**	*mat1Msmto leu1-32 ura4D18 ade6-210 his2*
**SPP192**	*mat1Msmto leu1-32 ura4D18 REII Δmat2::ura4 ade6-216 with YFP-FLAG-(his)6 tagged cut4 integrated at leu1 by digesting it with ApaI*
**SPP91**	*mat1PΔ17::LEU2 REII Δmat2P::ura4 leu1-32 ura4D18* $his2^-$ *ade6-210* $[SNG2]_P$
**SPP79**	*mat1Msmto REIIΔmat2 ura4 leu1-32 ura4D18 ade6-216* $clr3\Delta::kan^r$ *(dark)*
**SPP75**	*mat1Msmto leu1-32 ura4D18 REII ΔmatP2::ura4 ade6-216 hsp104Δ::* kar^r
**SPP76**	*mat1Msmto leu1-32 ura4D18 REII Δmat2P::ura4 ade6-216 cut4::cut4(GFP::*kan^r*)*
**SPP87**	*mat1Msmto leu1-32 ura4D18 REII Δmat2P::ura4 ade6-2160* $[SNG2]_{mat1M}$
**SPP180**	*mat1Msmto leu1-32 ura4D18 REII Δmat2P::ura4 ade6-216 tht1Δ::* kan^r<br>*mat1Msmto leu1-32 ura4D18 REII Δmat2P::ura4 ade6-210 tht1Δ::* kan^r
**SPP153**	*mat1PΔ17::LEU2 REII Δmat2P::ura4 leu1-32 ura4D18* $his2^-$ *ade6-216 tht1Δ::* kan^r
**SPP162**	*mat1PΔ17::LEU2 REII Δmat2P::ura4 leu1-32 ura4D18* $his2^-$ *ade6-216 tht1Δ::* $kan^r[SNG2]_P$
**SSP01**	$[SNG2]^O$ derivative generated by overexpression of *cut4* gene in strain *SPP75*
**SPP181**	*mat1Msmto leu1-32 ura4D18 REII Δmat2P::ura4 ade6-216 tht1Δ::* kan^r $[SNG2]_M$<br>*mat1Msmto leu1-32 ura4D18 REII Δmat2P::ura4 ade6-210 tht1Δ::* $kan^r cut4^+$
**SPP183**	*mat1PΔ17leu1-32 ura4D18 REII Δmat2P::ura4 ade6-210 tht1Δ::* kan^r $[SNG2]_P$<br>*mat1PΔ17leu1-32 ura4D18 REII Δmat2P::ura4 ade6-216 tht1Δ::* kan^r $cut4^+$
**SPP201**	*mat1Msmto leu1-32 ura4D18 REII Δmat2P::ura4 ade6-210 tht1Δ::* kan^r *swi6Δ::his1*<br>*mat1Msmto leu1-32 ura4D18 REII Δmat2P::ura4 ade6-210 tht1Δ::* kan^r
**SPP152**	*mat1Msmto leu1-32 ura4D18 REII Δmat2P::ura4 ade6-210 tht1Δ::* kan^r

Table S2. Plasmids used in the study

**Plasmid Name**	**Description**
pREP3X	*nmt1* promoter with *LEU2* marker S.pombe expression vector
pYY463	*cut4*$^{+}$ gene in pREP41 vector under *nmt1* promoter with *LEU2* gene as selectable marker (high copy expression vector)
pYY439	*cut4*$^{+}$ gene (14kb cosmid clone 813) in pYC11 integration vector with *LEU2* gene as selectable marker
pYY463-dB/B	Essentially pYY463 vector with a deletion of fragment 848-3124 between two BglII sites of *cut4* gene
pREP41HAN	*nmt1* promoter with *LEU2* marker in expression vector for HA tagging ofproteins in *S. pombe*
pREP41HAN Cut4	*nmt1* promoter with *LEU2* marker in expression vector containing HA-Cut4
pREP41HAN Cut4-dB/B	nmt1 promoter with *LEU2* marker in expression vector having truncated HA-Cut4 with a internal deletion at BglII sites
YFH-Cut4	Full length Cut4 plasmid with YFP-(his)6-FLAG tag at N-terminal of cut4procured form Riken DNA Bioresource Center

SUPPLEMENTARY FIGURE LEGENDS

Figure S1. Non-Mendelian segregation and mitotic stability of the spo+-ura+ phenotype of the [SNG2] prion form. (A) Table showing the score of ura^+ and spo^+ phenotypes in the cross shown in Figure 2. Top panel shows the segregation of ura^+:ura^w phenotype among the tetrads generated from the cross. Lower panel shows the score for the different combination of the ura^+ and spo^+ phenotypes among the ts^+ and ts^- segregants of the cross. **(B)** Mitotic stability of the phenotypic states displayed by the segregants of the cross shown in Figure 3B. The states D and L represent the spo^+ and spo^+ phenotypes and ura^+, ura^- and ura^w represent strong growth, no growth or weak growth on medium lacking uracil.

Figure S2I Mendelian segregation of phenotypes generated by a canonical heterochromatin mutant. (A) spo^--ura^- phenotype of the segregants generated from a cross between the WT parent strains: Tetrads derived from a cross between WT strains with genotype I (*mat1Msmto leu1-32 REIIΔmat2::ura4 ura4D18)* and genotype II *(mat1PΔ17:: LEU2 leu1-32 REIIΔmat2::ura4 his2 ura4D18),* were replica plated onto indicated plates. The genotypes are mentioned above and below the tetrad dissection panel. **(B)** Co-segregation of spo^+-ura^+ phenotypes with *clr3Δ* mutation during meiosis: Tetrad analysis of a cross between *clr3Δ::kan^r^* mutant with genotype I that gives spo+/ura+ phenotype and a wild type strain with genotype II. The genotypes are mentioned above and below the tetrad dissection panel.

Figure S3I de novo generation of [SNG2] prion-form by high level expression of *cut4* and its non-Mendelian segregation. (A) Serial dilution spotting assay of WT strain with genotype I,

transformed with empty vector, integrating vector containing intact *cut4* gene and truncated copy of the *cut4* gene (DB/B). **(B)** Western analysis of cell expressing the pREP41HAN vector alone (v), full length *cu4* gene (*cut4*) or truncated *cut4* gene (*cut4DB/B*). Cells were grown to express the genes under *nmt41* promoter, followed by SDS-PAGE (10%) and western blotting with monoclonal anti-HA antibody. * indicates the non-specific cross-reactive band, arrowhead indicates the truncated band of cut4 expressed by the vector expressing HA-Cut4 DB/B. **(C)** Results of segregation of spo+/ura+ phenotype in a backcross of the [SNG2]^{o} derivative obtained after loss of the *cut4* gene on a high copy plasmid, shown in Figure 4D from a strain with genotype I with a wt strain with genotype II.

Figure S4IAnalysis of prion characteristics of [SNG2]^{Cut4}

(A) **Curing of prion form by hsp104.** [SNG2] cell harboring GFP-Cut4 were cultured in the presence of empty vector or hsp104 gene under the control of nmt1 promoter, followed by SDDAGE analysis. The gel was run for half the normal length of the gel. **(B)** SDDAGE analysis of soluble and pellet fractions of normal strain expressing GFP-Cut4 and Light and dark derivatives of [SNG2]$^{GFP\text{-}Cut4}$. Arrows indicate the oligomeric forms of Cut4. **(C)** Slot blot analysis of fractions shown in (B).

Figure S5I Lack of intrachromosomal recombination and interchromosomal segregation in a strain with a homozygous karyogamy mutation *tht1Δ/tht1Δ*. Strain having linked leu^{+}(*mat1P*-linked)/*his2*$^{-}$ on chrII along with an *ade6-210* allele on chromosome III was crossed with another *tht1Δ* strain having *mat1*-linked *leu1*$^{-}$/*his2*$^{+}$ alleles on chromosome II and *ade6-216* allele on chromosome III and subjected to random spore analysis followed by scoring of the *leu1*, *his2* and *ade6* markers. Results show no

cross over between *leu* and *his2* loci and show co-segregation of chr II and III.

Figure S6I Dominant negative effect of [SNG2] prion-form on silencing at *ade6* reporter inserted at the centromere and *mat3* loci. **(A)** Schematic representation of the centromere I, showing central element *cnt1*, the inner repeats *imr1L* and *imr1R* and outer repeats *otr1L* and *otr1R*. Also shown are insertion of *ura4* and *ade6* reporter at *imr1R* and *otr1R* repeat region, respectively. WT strains wherein both the reporters are silent, display low growth and red colored colonies on plates lacking uracil or limiting amount of adenine, respectively. **(B)** Organization of the mating type locus, showing *mat1P* locus having a deletion of cis-acting region along with a *LEU2* reporter insertion (*mat1PΔ17::LEU2*) and *mat3*-linked *ade6* reporter. WT strains form red colored colonies on media containing limiting adenine. Loss of silencing in (A) and (B) leads to pink/white phenotype of colonies on adenine limiting plates. **(C)** 5 random putative prion$^+$ segregants having *mat1M* allele labelled "$[SNG2]_M$"1-5 were obtained from the cross of $[SNG2]_P$ with *mat1Msmto* strain (SPJ25) and a normal *mat1Msmto* strain (SPJ25). These were further crossed with a strain containing stable *mat1P* locus and having *ade6* inserted at the *otr1* region of centromere **(A, panel a)** or *mat3* locus **(B, panel b).** Random spores from the crosses (1-5), along with a control *Msmto* strain (SPJ25) were streaked on adenine limiting plate (YE), grown at 30°C for 4 days and photographed.

Figure S7I Overexpression of *cut4* abrogates silencing at the *ade6* reporter inserted at the *otr1R* locus. Strain having ade6 insertion at the *otr1R* site on *chrI* was transformed with high copy vector *pREP3* and the same vector containing the *cut4* gene or a

copy of *cut4* gene having internal deletion of the *Bgl*II-*Bgl*II region (cut4DB/B). Transformants were streaked on selective plates having low amount of adenine. After 3-4 days' growth at 30°C, the colonies were counted and photographed.

Figure S8I Delocalization of Swi6 and H3-K9-me2 from silent *mat2* locus in the [SNG2] cells.

(A) Schematic representation of the mating type locus, showing the stable *mat1* locus *mat1Msmto*, *mat2P* locus with cis-acting deletion *REIIΔ* and linked *ura4* reporter and *mat3M* locus. (B) ChIP experiment was performed to measure the localization of Swi6 and H3K9-Me2 at the *ura4* reporter. The upper band represents the *ura4* reporter inserted distally to the *mat2P* locus, while the smaller band represents the truncated *ura4DS/E* locus present at the normal location of *ura4*. **(B)** ChIP assay was performed to quantitate the localization of Swi6 and H3-K9-Me2 at the *mat2*-linked *ura4* reporter in the indicated strains, using the euchromatic *ura4DS/E* as a control. Ratio of PCR signal at the ura4 and *uraDS/E* for the IP versus the whole cell extract (WCE) is given as enrichment ratio. **(C)** The enrichment ratios shown in **(B)** are plotted.

Figure S9I Effect of prion form of Cut4 on survival from ethanol stress. (A) Plate assay performed for normal and prionic strains with and without guanidine treatment. Cultures were spotted on normal YEA plates and plates containing ethanol gradient (0-7%). **(B)** Growth kinetics of the strains shown in **(A)**, grown in presence and absence of guanidine in presence of 0, 2% and 3% ethanol.

Figure S10I PSIPRED analysis of Cut4 sequence. The amino acid sequence of Cut4 was subjected to analysis for prediction of secondary structure (PSIPRED) (**A**) and IDR using DISOPRED3

(B). Regions with high propensity to adopt Intrinsically Disordered Structure.

A

Ratio ura$^+$: ura^w	No. tetrads
4:0	9/ 54 (16%)
3:1	12/ 54 (22%)
2:2	23/ 54 (41%)
1:3	6/ 54 (11%)
0:4	4/ 54 (7%)

Total ascospores	216
ura$^+$	124:<br>63 his+<br>61his-
ura$^-$	92
ts+ ura+	64 (30%)
ts+ ura-	40 (18%)
ts- ura+	60 (29%)
ts- ura-	52 (23%)
ts+ spo+	34 (16%)
ts+ spo-	70 (32%)
ts- spo+	47 (22%)
ts- spo-	65 (30%)

B

	Nature of switch	rate of switching of spo phenotype
DSPR	D to L	$2.7X10^{-4}$
LSPR	L to D	$1.9X10^{-4}$
Msmto REIIΔmat2::ura4	L to D	$1.05X10^{-5}$
[SNG2] spo$^+$-ura$^+$	D to L	$5.6X10^{-5}$
[SNG2] spo$^-$-ura$^+$	L to D	$1.7X10^{-4}$
[SNG2] spo$^+$-ura^w	D to L	$2.3X10^{-5}$
[SNG2]$_M$ spo^w-ura$^+$	L to D	$2.1x10^{-4}$

	Nature of switch	rate of switching of ura phenotype
DSPR	ura$^+$ to ura$^-$	$2.8X10^{-4}$
LSPR	ura$^+$ to ura$^-$	$3.9X10^{-4}$
Msmto REIIΔmat2::ura4	ura$^-$ to ura$^+$	$2.8X10^{-3}$
[SNG2]ura$^+$spo$^+$	ura$^+$ to ura$^-$	$3.2X10^{-4}$
[SNG2]ura$^+$spo$^-$	ura$^+$ to ura$^-$	$1.00X10^{-4}$
[SNG2]ura^{w}spo$^+$	ura^w to ura$^+$	$9X10^{-5}$
[SNG2]M ura$^+$spo^w	ura$^+$ to ura$^-$	$9.5X10^{-6}$

Supplementary Figure S1

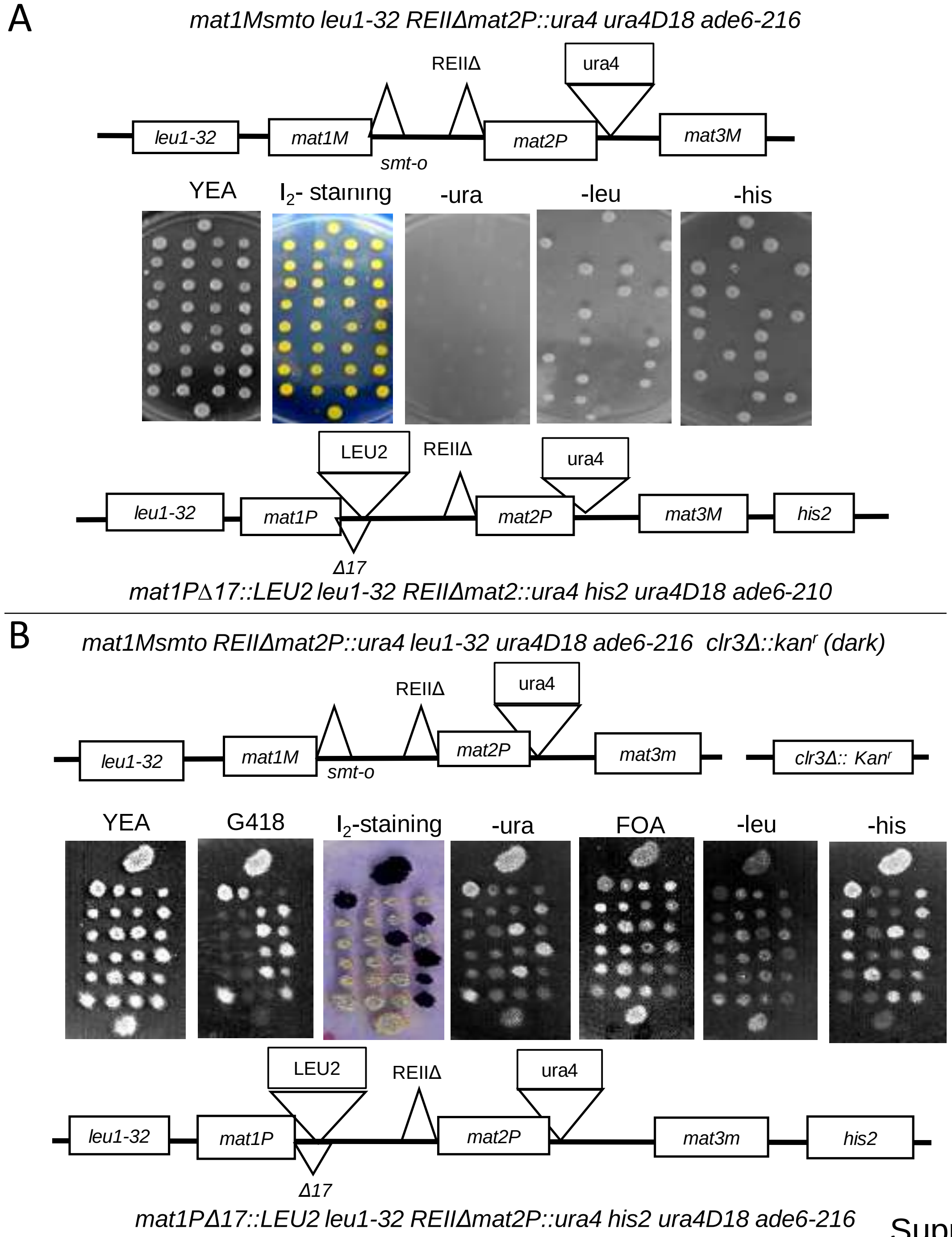

Supplementary Figure S2

A

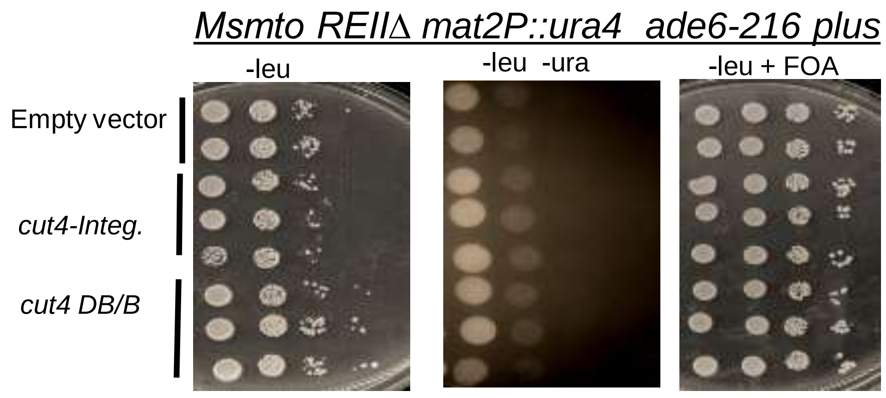

B

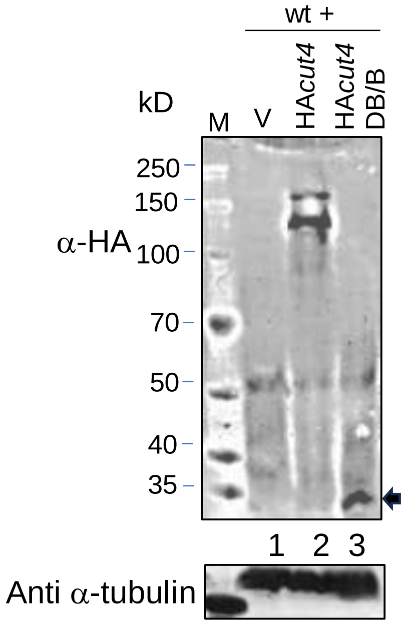

*

C

Ratio of ura⁺: ura^w	No. of tetrads (%)	ura⁺ leu⁺ his⁺	ura⁺ leu⁻ his⁺	ura⁺ leu⁺ his⁻
4:0	7/50 (14)	8	12	8
3:1	29/50(58)	26	40	21
1:3*	1/50 (2)	1	0	0
2:2	13/50 (26)	6	10	10

Supplementary Figure S3

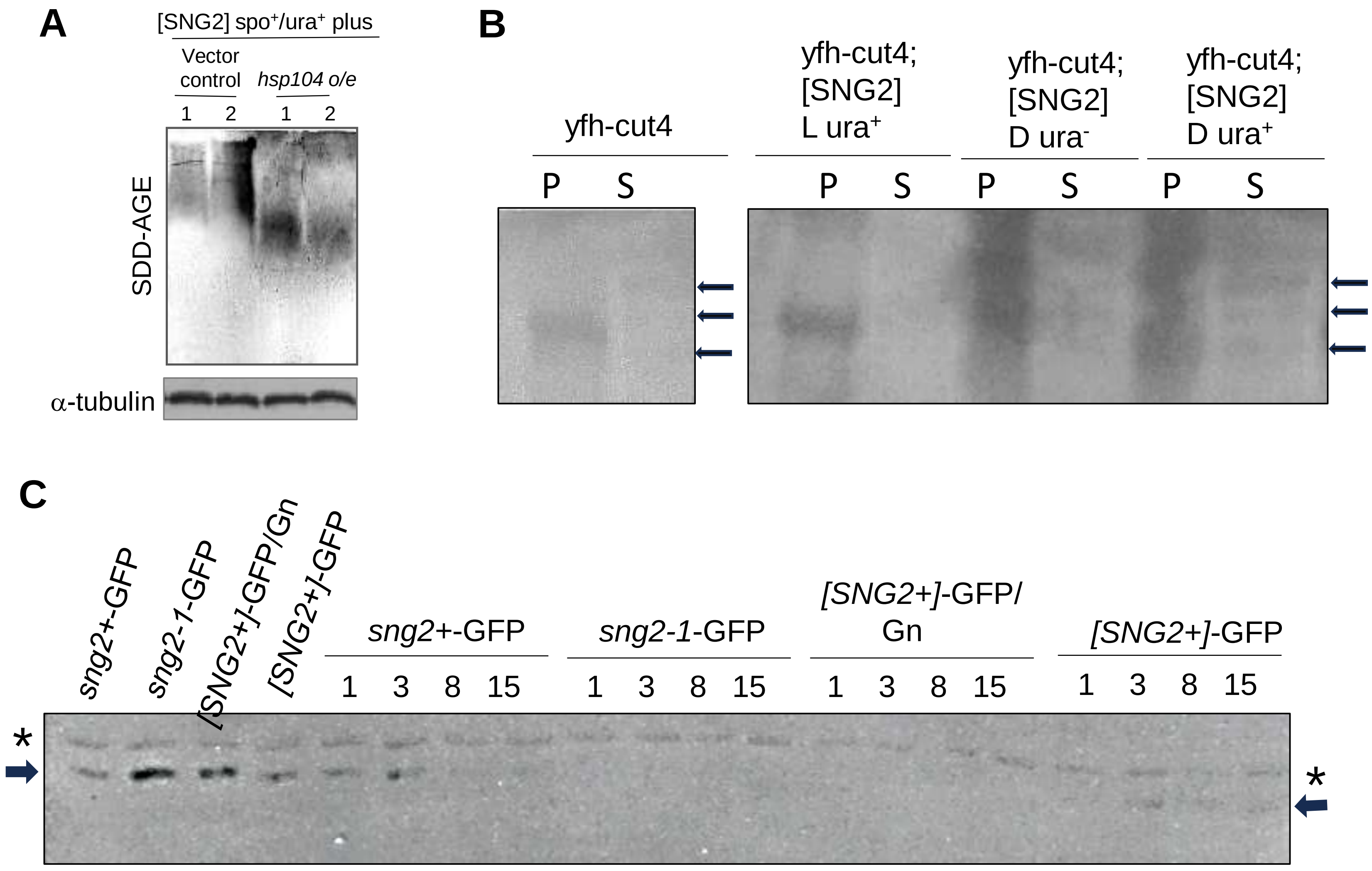

Supplementary Figure S4

<table>
<tr><td colspan="4">Msmto leu1-32 ura4D18 REII Δmat2P::ura4 ade6-216 tht1Δ::kan^{r}
X
PΔ17::LEU2 REII Δmat2P::ura4 leu1-32 ura4D18 his2^{-} ade6-210 tht1Δ::Kan^{r}
(Total no. of spores N= 276)</td></tr>
<tr><td rowspan="2">leu^{+} -his^{-}</td><td rowspan="2">117</td><td>ade6-210</td><td>117</td></tr>
<tr><td>ade6-216</td><td>0</td></tr>
<tr><td rowspan="2">leu^{--}-his^{+}</td><td rowspan="2">159</td><td>ade6-210</td><td>0</td></tr>
<tr><td>ade6-216</td><td>159</td></tr>
<tr><td>leu^{-} -his^{-}</td><td>0</td><td colspan="2">-</td></tr>
<tr><td>leu^{+}-his^{+}</td><td>0</td><td colspan="2">-</td></tr>
</table>

Supplementary Figure S5

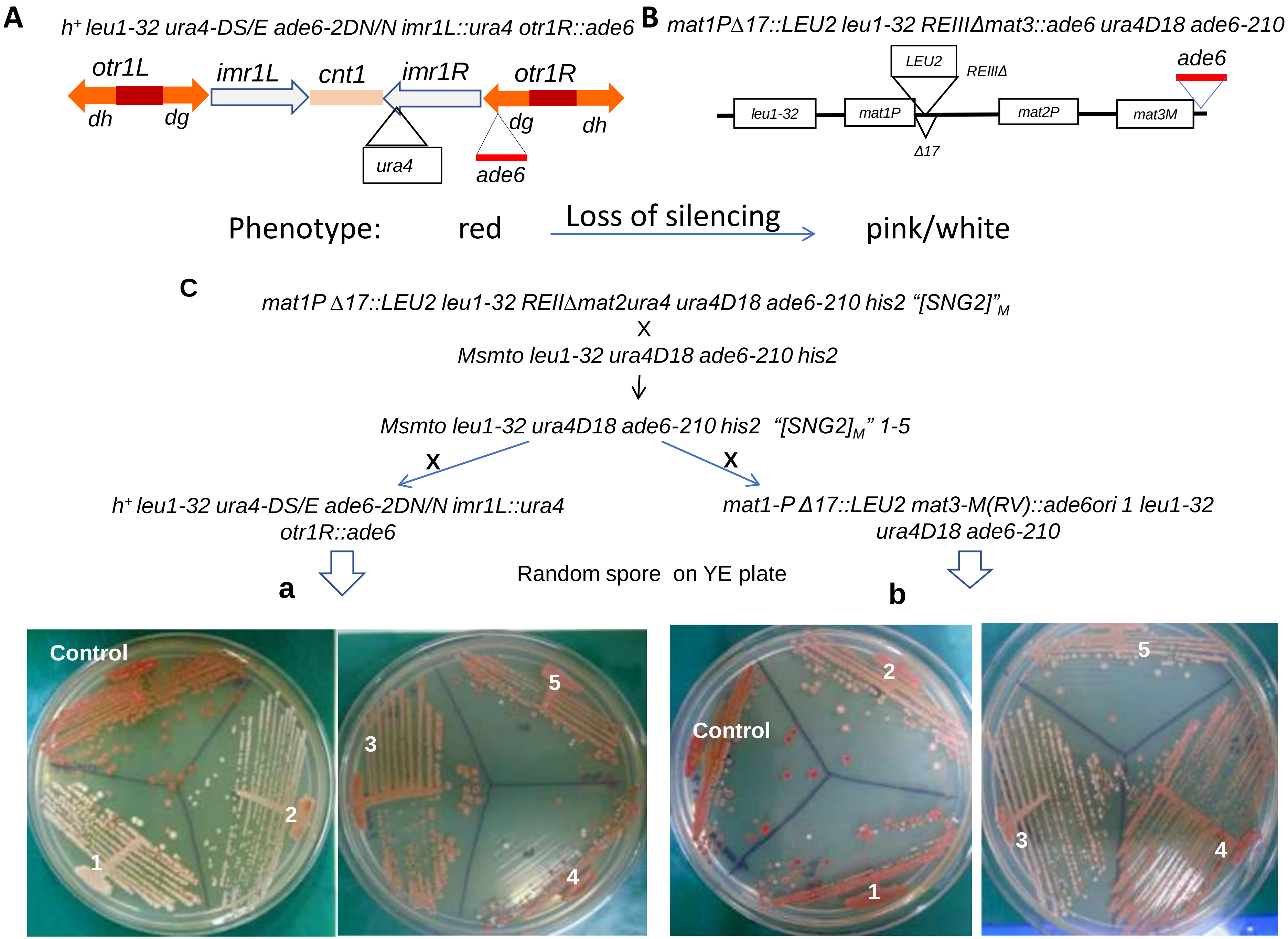

Supplementary Figure S6

otr1R plus

	pREP3	*cut4*	*cut4* DB/B

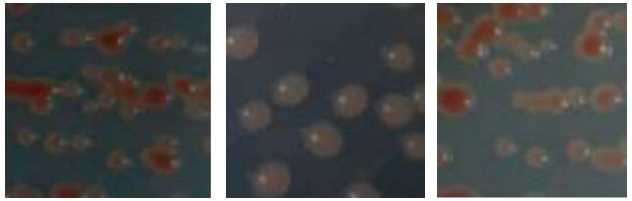

% Pink	11	35.4	19.8
% white	1.9	39.2	0

Supplementary Figure S7

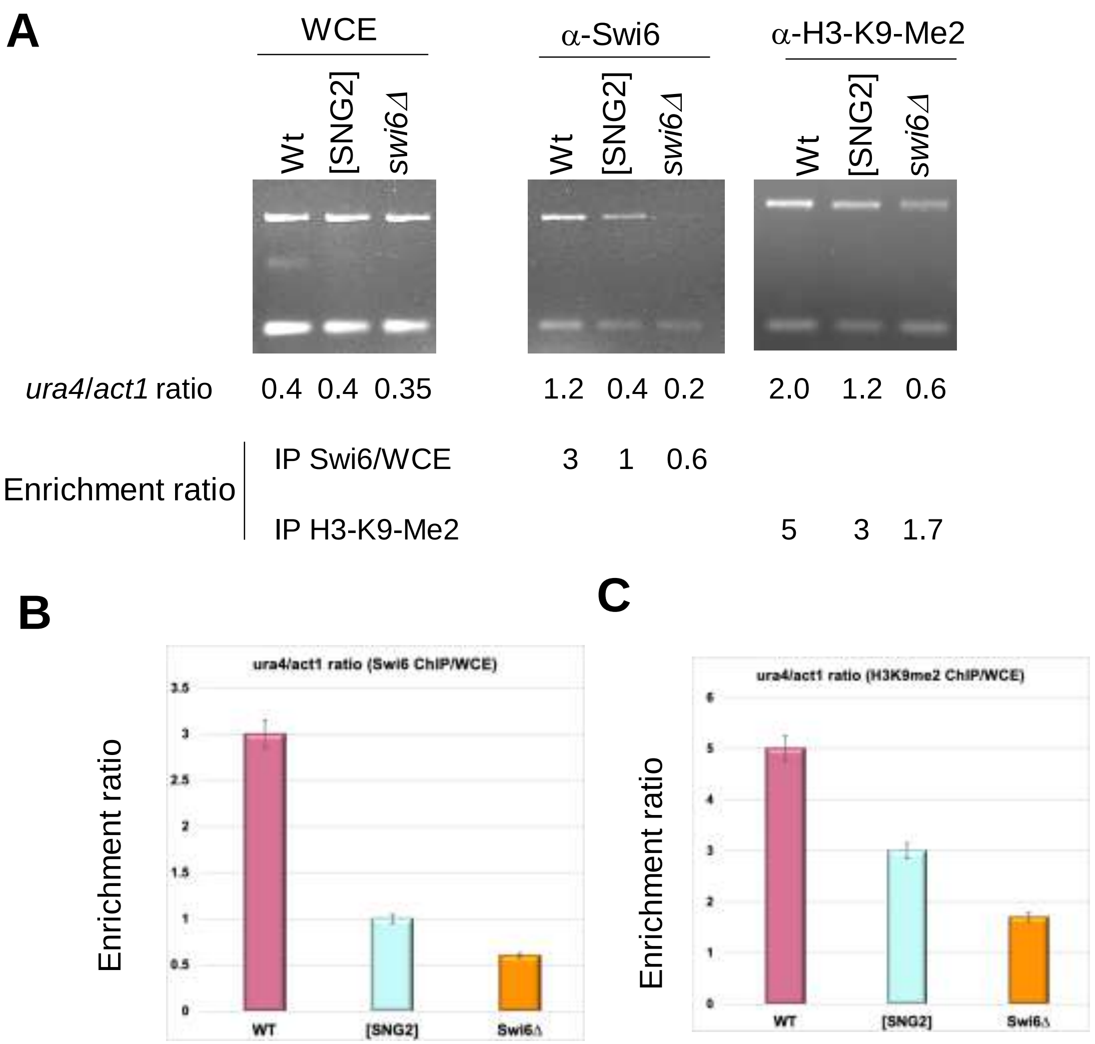

Supplementary Fig. S8

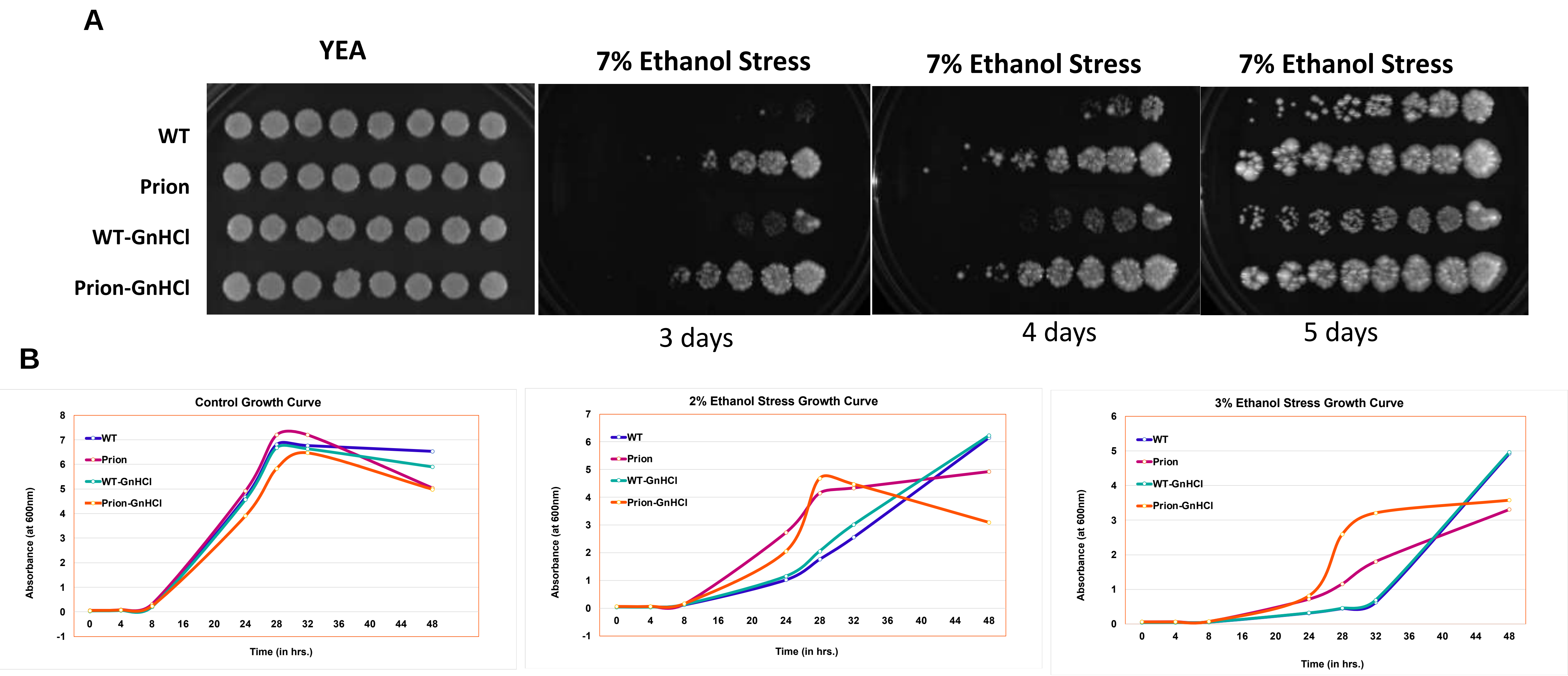

Supplementary Fig. S9

A

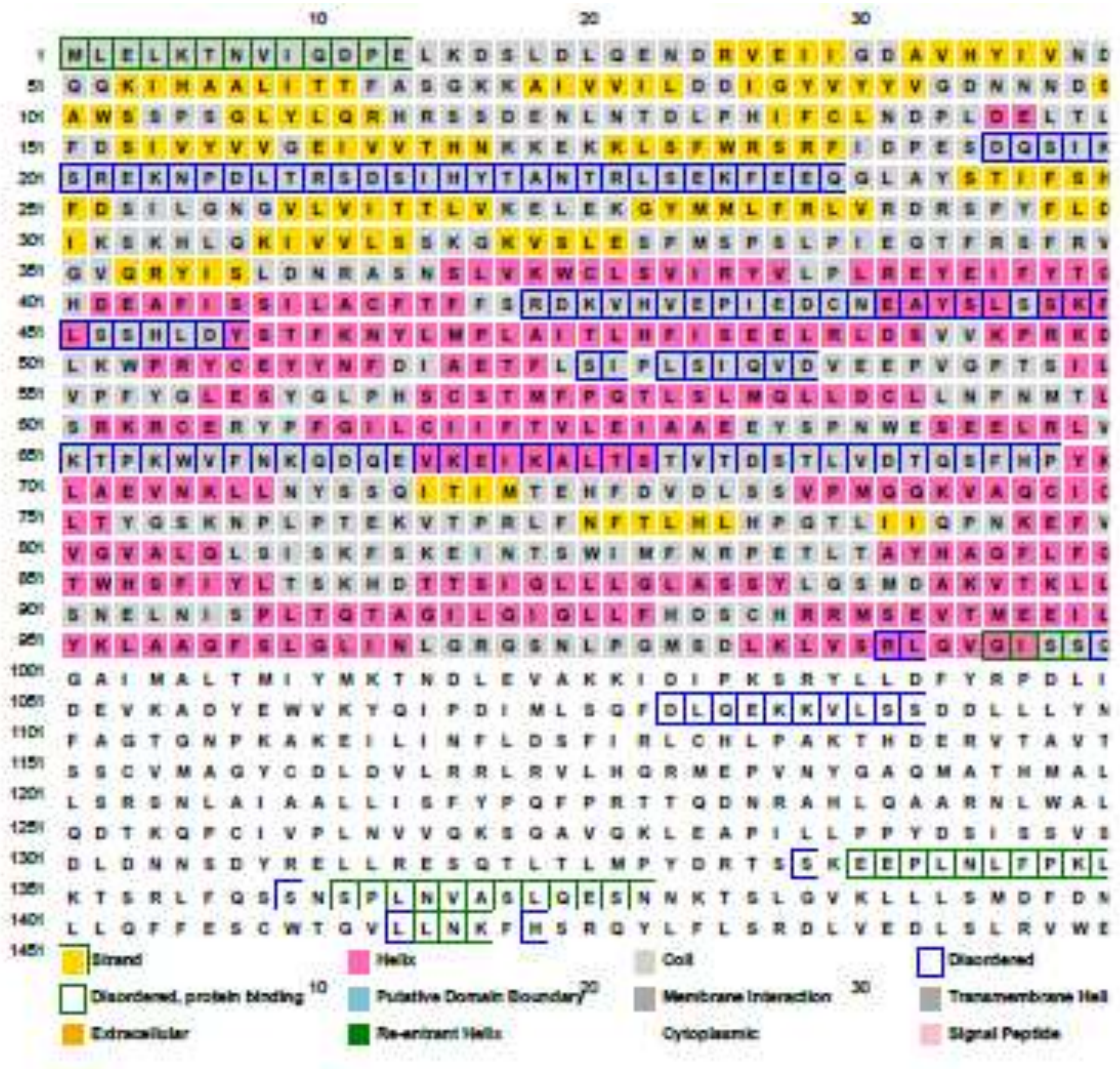

B

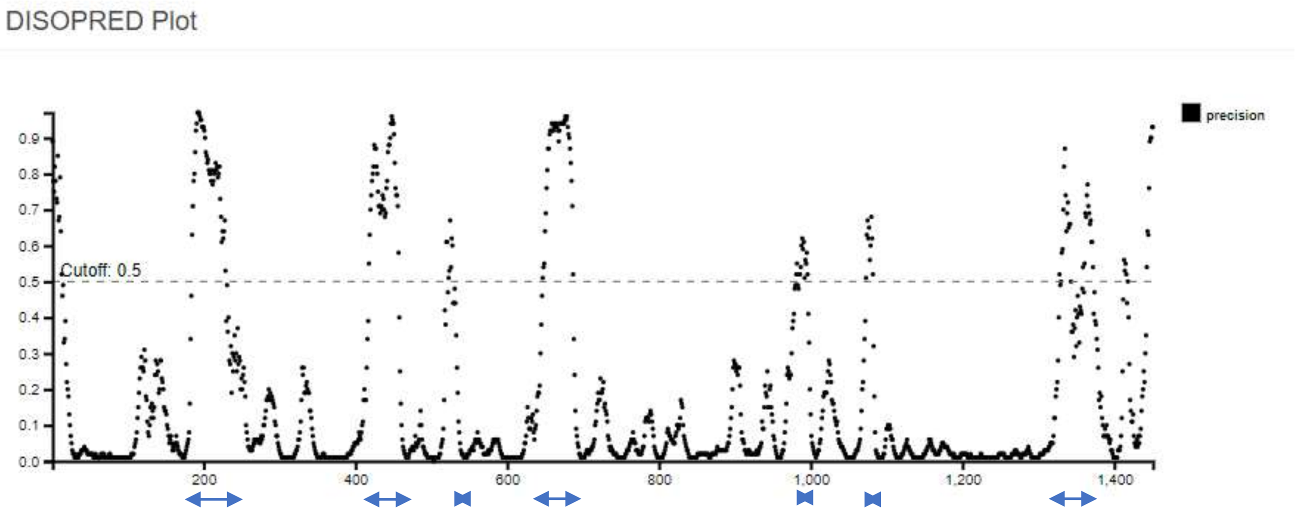

Supplementary Fig. S10
